# CD36 Regulates PANoptosis in Diabetic Retinopathy via the NOTCH/MAML Pathway

**DOI:** 10.1155/jdr/9324498

**Published:** 2026-07-24

**Authors:** Rongfeng Dai, Yu Qian, Siqi Liu, Yuqing You, Yan Han, Yi Fang

**Affiliations:** ^1^ Department of Endocrinology, Changzhou Third People′s Hospital, Changzhou, Jiangsu, China

**Keywords:** CD36, diabetic retinopathy, MAML, NOTCH, PANoptosis, retinal endothelial cells

## Abstract

Worldwide, diabetic retinopathy (DR) stands as a leading cause of vision loss. However, the involvement of PANoptosis—a form of inflammatory cell death that combines features of apoptosis, pyroptosis, and necroptosis—in the development of DR has not been fully elucidated. This study investigated the molecular mechanisms underlying high glucose (HG)–induced PANoptosis in human retinal microvascular endothelial cells (hRMECs), focusing on the scavenger receptor CD36 and NOTCH/MAML signaling. HG specifically induced PANoptosis in hRMECs, evidenced by concurrent activation of apoptotic, pyroptotic, and necroptotic markers, along with PANoptosome complex formation and morphological validation via terminal deoxynucleotidyl transferase dUTP nick end labeling (TUNEL) staining. HG significantly upregulated CD36 expression and activated the NOTCH/MAML pathway. CD36 overexpression exacerbated PANoptosis by enhancing cell death, inflammatory responses, and oxidative stress, whereas CD36 knockdown conferred protection. Mechanistically, CD36 promoted PANoptosome assembly through NOTCH/MAML pathway activation, as demonstrated by increased NICD‐MAML1 nuclear colocalization and enhanced NOTCH component expression. We further verified that the CD36‐NOTCH axis regulates PANoptosis through the modulation of NLRP3, a core component of the PANoptosome. Pharmacological NOTCH inhibition using DAPT ameliorated HG‐induced PANoptosis, whereas NOTCH activation mimicked CD36 overexpression effects. These results establish a novel CD36‐NOTCH/MAML‐NLRP3‐PANoptosis regulatory pathway in diabetic retinal endothelial cells. This discovery provides crucial insights into DR pathogenesis and pinpoints potential targets for therapeutic intervention.

## 1. Introduction

As a debilitating microvascular complication of diabetes, diabetic retinopathy (DR) is a leading cause of preventable blindness among the global working‐age population [1, 2]. Its prevalence is rising with the diabetes pandemic, which impacts over 500 million individuals globally [3]. Current treatments, including laser photocoagulation and anti‐VEGF agents, target late‐stage disease and have limited efficacy [4, 5]. This underscores the need to unravel early stage DR molecular pathways. DR pathophysiology is a multifactorial process driven by chronic hyperglycemia, which triggers oxidative stress, inflammation, and metabolic reprogramming [6, 7]. These insults induce dysfunction and death of retinal cells, including RPE cells, endothelial cells, and pericytes, compromising the blood–retinal barrier [8, 9]. Understanding diverse cell death modalities is fundamental to decoding DR pathology.

Distinct from classical apoptosis, PANoptosis is a novel and highly inflammatory type of programmed cell death. It mechanistically integrates pyroptosis, apoptosis, and necroptosis, driven by the PANoptosome complex [10, 11]. Recent studies have confirmed the existence of PANoptosis‐like cell death in ischemia/reperfusion injury of retinal neurons [12], as well as in cerebral ischemia [13], indicating its critical role in central nervous system diseases. While PANoptosis has been implicated in diabetic nephropathy and cardiomyopathy [14, 15], its role in DR remains unexplored.

CD36, a transmembrane glycoprotein of the Class B scavenger receptor family, is involved in fatty acid transport, endothelial regulation, inflammation, oxidative stress, and apoptosis [16]. In the diabetic retina, CD36 upregulation promotes lipid deposition, reactive oxygen species (ROS) production, and inflammatory cytokine secretion, processes that induce both apoptosis and vascular barrier disruption [17]. Recent machine learning analyses have identified CD36 as a potential core gene in DR‐associated PANoptosis, although its precise signaling role remains unclear [18]. Furthermore, CD36 has been shown to coordinate NLRP3 inflammasome activation by facilitating intracellular nucleation of soluble ligands in sterile inflammation [19], positioning it as a central regulator of inflammasome activation. As a highly conserved signaling pathway, NOTCH orchestrates critical cell fate decisions. Its downstream Mastermind‐like (MAML) proteins are key coactivators for the Notch intracellular domain (NICD)–dependent transcription [20]. NOTCH pathway dysregulation in the diabetic retina is linked to inflammation and vascular dysfunction [21]. Aberrant activation of NOTCH1 signaling, for instance, has been shown to induce pathological vascular permeability, a hallmark of diabetic macular edema [22]. Moreover, NOTCH signaling depends on MAML coactivators to upregulate genes that promote cell survival [23]. However, whether a direct signaling axis connects the upstream receptor CD36 to the downstream execution of PANoptosis in diabetic retinal cells, and the potential involvement of the NOTCH pathway in bridging this gap, remains a significant and unanswered question in the field.

Based on this background, this study investigates the potential “CD36‐NOTCH/MAML‐NLRP3‐PANoptosis” regulatory axis. Utilizing an in vitro high glucose (HG)–induced retinal cell model alongside molecular and cytopathological techniques, we will characterize CD36 expression changes during retinal PANoptosis in DR, verify its regulatory role on key NOTCH/MAML pathway molecules, and elucidate the specific mechanism through which this pathway mediates retinal PANoptosis. The findings from this study seek to advance the theoretical understanding of DR pathology, thereby offering experimental validation for novel interventions that target CD36 or NOTCH/MAML signaling.

## 2. Materials and Methods

### 2.1. Cell Culture and Pretreatment

Human retinal microvascular endothelial cells (hRMECs) obtained from Procell Biotechnology (China) were cultured under standard conditions: RPMI 1640 medium (Thermo Fisher, United States) with 10% FBS and 1% penicillin–streptomycin, at 37°C in a 5% CO_2_ humidified incubator.

In experiments, cells were assigned to three treatment groups for 72 h: the normal glucose (NG) group with 5.5 mmol/L D‐glucose, the HG group with 25.0 mmol/L D‐glucose, and the osmotic control (OC) group with 5.5 mmol/L D‐glucose and 19.5 mmol/L D‐mannitol to control for osmotic effects. All experiments were performed with at least three independent biological replicates (*n* ≥ 3).

### 2.2. Gene Overexpression and Knockdown

CD36 overexpression and knockdown were achieved through transfection using Lipofectamine 3000 (Thermo Fisher, United States). Specifically, cells were transfected with either a pcDNA3.1‐CD36 plasmid (for overexpression) or an empty pcDNA3.1 vector (OE‐NC control). For knockdown, a specific siRNA targeting CD36 (si‐CD36) (Santa Cruz Biotechnology, United States) or a nontargeting control siRNA (si‐NC) was used. All procedures followed the manufacturer′s instructions.

The experimental procedure was as follows: Cells were first divided into six groups. The OE‐CD36 and OE‐NC groups were transfected with a CD36‐overexpressing plasmid and an empty vector, respectively. Concurrently, the si‐CD36 and si‐NC groups were transfected with CD36‐targeting siRNA and a nontargeting control siRNA, respectively. Following this transfection step, all groups, except the NG group (which was kept in 5.5 mmol/L D‐glucose), were cultured in medium containing 25.0 mmol/L D‐glucose for 72 h. The HG group received the HG treatment without prior transfection.

### 2.3. NOTCH Pathway Modulation

To modulate NOTCH signaling under HG conditions, cells were cultured in 25.0 mmol/L D‐glucose for 72 h. During the final 24 h, the HG group was supplemented with either 5 *μ*g/mL Jagged1 peptide (MedChemExpress, United States) to activate the pathway or 10 *μ*M DAPT (MedChemExpress, United States) to inhibit it. An equivalent volume of DMSO served as the solvent control. The NG group was maintained in 5.5 mmol/L D‐glucose throughout.

### 2.4. Cell Viability Assay

The Cell Counting Kit‐8 (CCK‐8; Beyotime, China) was used to determine cell viability. After treatment, cells (seeded at 5 × 10^3^ cells/well in a 96‐well plate) were incubated with 10 *μ*L CCK‐8 solution for 2 h at 37°C, and the absorbance was read at 450 nm using an RT‐6000 microplate reader (Rayto, China).

### 2.5. Analysis of Apoptosis by Flow Cytometry

Apoptosis was determined by flow cytometry using an Annexin V‐FITC/PI detection kit (Beyotime, China). Treated cells were harvested, washed with cold PBS, and resuspended in 1X binding buffer. Subsequently, they were subjected to dual staining with 5 *μ*L Annexin V‐FITC and 5 *μ*L PI for 15 min in the dark at room temperature. Analysis was conducted on an EVOS M5000 flow cytometer (Thermo Fisher, United States) within 1 h of staining.

### 2.6. Terminal Deoxynucleotidyl Transferase dUTP Nick End Labeling (TUNEL) Assay

To evaluate the morphological features of cell death, TUNEL staining was performed using a TUNEL Apoptosis Assay Kit (Beyotime, China) according to the manufacturer′s instructions. Cells cultured on coverslips were fixed with 4% paraformaldehyde for 30 min, permeabilized with 0.3% Triton X‐100 for 5 min, and incubated with the TUNEL reaction mixture for 1 h at 37°C in the dark. Nuclei were counterstained with DAPI. Images were captured using an Eclipse Ti‐E fluorescence microscope (Nikon Corporation, Japan) at 20x magnification, and the apoptosis rate was calculated as the percentage of TUNEL‐positive cells relative to total DAPI‐stained nuclei from at least five randomly selected fields per sample.

### 2.7. ELISA

The concentrations of IL‐1*β*, IL‐18, TNF‐*α*, and IL‐6 in the cell culture supernatants were measured using commercial ELISA kits (Lianke Biotechnology, China) according to the manufacturer′s protocols. Absorbance was measured at 450 nm.

### 2.8. ROS Measurement

According to the manufacturer′s instructions, intracellular ROS was detected with the DCFH‐DA probe (Enzyme‐linked, China). Treated cells were incubated with 10 *μ*M DCFH‐DA for 30 min at 37°C prior to fluorescence intensity measurement on an EVOS M5000 flow cytometer (Thermo Fisher, United States).

### 2.9. Caspase‐3/7 Activity

Caspase‐3/7 activity was determined with the Caspase‐Glo 3/7 Assay Kit (Beyotime, China). Treated cells in 96‐well plates were lysed with the reagent and incubated for 1 h at room temperature in the dark. The resulting luminescence was quantified and normalized to cell viability data.

### 2.10. qPCR

According to the manufacturer′s instructions, total RNA was isolated using TRIzol reagent (Tiangen Biochemical, China) and subsequently reverse‐transcribed into cDNA (PrimeScript RT Reagent Kit; Takara, China). Real‐time qPCR was conducted on an ABI 7500 Detection System (Applied Biosystems, United States) using SYBR Green Master Mix (Yeasen Biotechnology, China). Relative gene expression levels were determined by the 2^−*ΔΔ*Ct^ method using GAPDH as the endogenous control. All primer sequences are listed in Supporting Information 4: Table S1.

### 2.11. Western Blotting

Proteins were extracted with RIPA lysis buffer (Beyotime, China) and quantified via a BCA assay (Yeasen Biotechnology, China). Equal amounts of protein lysates were resolved by SDS‐PAGE, transferred to PVDF membranes, and blocked with 5% nonfat milk. The membranes were then probed overnight at 4°C with primary antibodies against cleaved Caspase‐3, Bax, Bcl‐2, GSDMD‐N, RIPK1, RIPK3, phosphorylated MLKL (p‐MLKL), CD36, Notch1/2/3, NICD, and MAML1/2/3 and NLRP3 (see Supporting Information 4: Table S2 for antibody catalog numbers and dilution ratios). After incubation with an HRP‐conjugated secondary antibody, bands were detected, and their gray values were analyzed with ImageJ software. The uncropped Western blot membranes for all blots presented in this manuscript are provided in Supporting Information 5: Figures S3–S8.

### 2.12. Immunofluorescence (IF)

Cells on glass coverslips were fixed with 4% paraformaldehyde, permeabilized with 0.1% Triton X‐100, and blocked with 5% BSA. They were then incubated overnight at 4°C with primary antibodies against CD36, NICD, MAML1, Caspase‐1, ASC, or p‐MLKL (see Supporting Information 4: Table S3). After washing, corresponding Alexa Fluor–conjugated secondary antibodies were applied for 1 h. Nuclei were counterstained with DAPI prior to image acquisition using an Eclipse Ti‐E fluorescence microscope (Nikon Corporation, Japan).

### 2.13. Coimmunoprecipitation (Co‐IP)

Co‐IP was performed using nondenaturing cell lysates. After preclearing with Protein A/G PLUS‐Agarose beads (Santa Cruz Biotechnology, United States), the lysates were immunoprecipitated overnight at 4°C with antibodies targeting Caspase‐1, ASC, NLRP3, RIPK3, or a control IgG (Supporting Information 4: Table S3). The complexes were pulled down by additional Protein A/G beads for 4 h and washed, and the eluted proteins were subjected to Western blot analysis.

### 2.14. Statistical Analysis

Data from at least three independent experiments (*n* ≥ 3) are expressed as mean ± SD. The normal distribution of data was assessed using the Shapiro–Wilk test. Statistical significance was determined using one‐way ANOVA followed by Tukey′s post hoc test for multiple comparisons in GraphPad Prism 8.0, with a *p* value < 0.05 considered significant.

## 3. Results

### 3.1. HG Induces Decreased Viability and Characteristic Activation of PANoptosis in Retinal Cells

**Figure 1:**
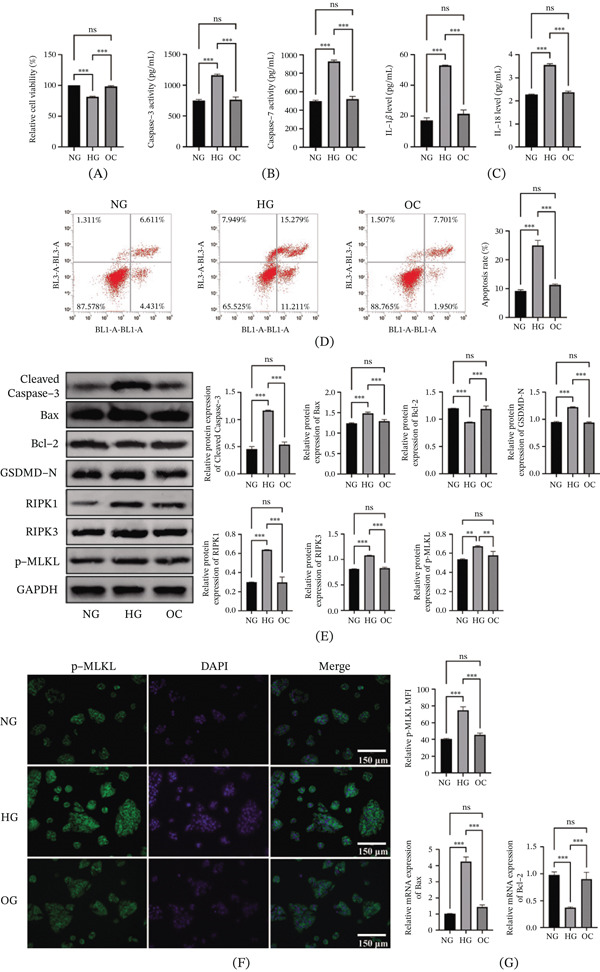
HG induces PANoptosis in hRMECs. (A) Cell viability of hRMECs detected by CCK‐8 assay. (B) Caspase‐3 and Caspase‐7 activities measured by colorimetric assay. (C) Assessment of secreted IL‐1*β* and IL‐18 via ELISA. (D) Assessment of apoptosis by Annexin V‐FITC/PI flow cytometry. (E) Western blot analysis of cleaved Caspase‐3, GSDMD‐N, RIPK1, Bax, RIPK3, p‐MLKL, and Bcl‐2 protein levels with quantification. (F) IF staining of p‐MLKL and quantification of mean fluorescence intensity (MFI). Scale bar = 150 *μ*m. (G) qPCR analysis of *Bax* and *Bcl-2* mRNA levels. Data are presented as mean ± SD (*n* = 3). Statistical analysis was performed using one‐way ANOVA followed by Tukey′s post hoc test.  ^∗∗^ *p* < 0.01,  ^∗∗∗^ *p* < 0.001, ns = not significant.

To model hyperglycemia‐specific effects, hRMECs were exposed to NG, HG, or an OC with mannitol for 72 h. As shown in Figure 1A, CCK‐8 assay results indicated that cell viability was significantly reduced in the HG group compared with the NG group (*p* < 0.001), whereas the OC group showed no significant change, suggesting that the damage was caused by HG rather than hyperosmolarity. The apoptosis rate was significantly higher in the HG group than in the NG and OC groups, as quantified by flow cytometry (Figure 1D, *p* < 0.001). This finding was further supported by a concurrent marked increase in the activity of the key apoptotic enzymes Caspase‐3/7 (Figure 1B, *p* < 0.001). Furthermore, we examined markers for three distinct programmed cell death pathways: apoptosis, pyroptosis, and necroptosis. As shown by Western blotting (Figure 1E), the HG group exhibited a significant upregulation of cleaved Caspase‐3 and the proapoptotic protein Bax, in contrast to a concurrent reduction in the antiapoptotic protein Bcl‐2 (*p* < 0.001). This was corroborated at the transcriptional level by qPCR, which showed increased *Bax* mRNA and decreased *Bcl-2* mRNA (Figure 1G, *p* < 0.001). For pyroptosis, the HG group showed a significant increase in the GSDMD‐N terminal fragment, accompanied by elevated secretion of the inflammatory cytokines IL‐1*β* and IL‐18 (Figure 1C,E; *p* < 0.001). Markers for necroptosis, including RIPK1, RIPK3, and p‐MLKL, were also significantly upregulated in the HG group (Figure 1E, *p* < 0.01). IF staining confirmed the increased expression and membrane translocation of p‐MLKL under HG conditions (Figure 1F).

To further confirm the morphological characteristics of cell death, we performed TUNEL staining. As shown in Supporting Information 1: Figure S1, the proportion of TUNEL‐positive cells with fragmented DNA was significantly elevated in the HG group compared with the NG group (*p* < 0.001), providing direct morphological evidence for the execution of apoptotic cell death programs.

**Figure 2:**
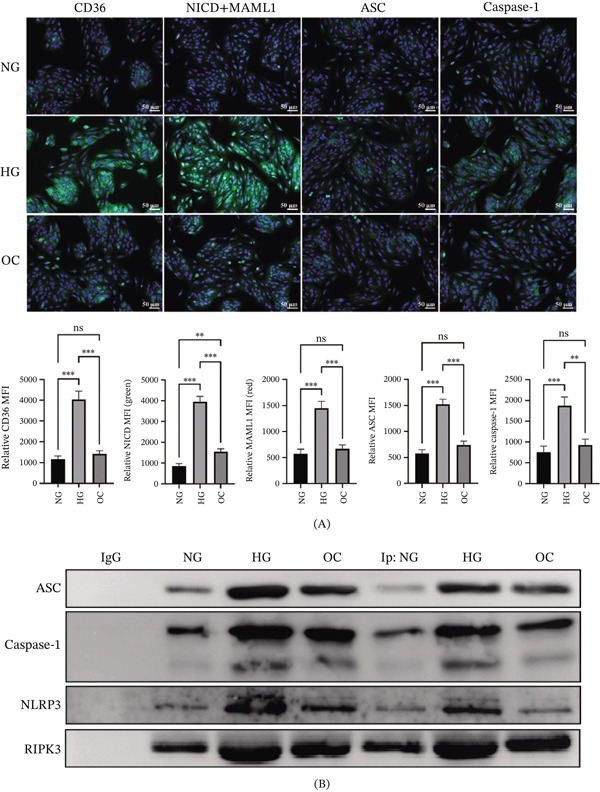
HG promotes PANoptosome formation and activates key signaling molecules. (A) Representative IF images and quantification showing CD36 expression (green), NICD + MAML1 colocalization (green + red), ASC (green), and Caspase‐1 (green). Nuclei were counterstained with DAPI (blue). Scale bar = 50 *μ*m. (B) Co‐IP analysis showing the interaction between ASC, Caspase‐1, NLRP3, and RIPK3, indicating the formation of the PANoptosome complex. Data are presented as mean ± SD (*n* = 3). Statistical analysis was performed using one‐way ANOVA followed by Tukey′s post hoc test.  ^∗∗^ *p* < 0.01,  ^∗∗∗^ *p* < 0.001, ns = not significant.

To further characterize the PANoptotic response, we examined the formation of the PANoptosome, a multiprotein complex that integrates apoptotic, pyroptotic, and necroptotic signaling. The formation of ASC and Caspase‐1 puncta, a hallmark of PANoptosome assembly, was notably enhanced in the HG group compared with controls, as revealed by IF analysis (Figure 2A, *p* < 0.001). Co‐IP experiments further confirmed the enhanced physical interaction among PANoptosome components, including ASC, Caspase‐1, NLRP3, and RIPK3, in the HG group (Figure 2B), indicating the assembly of a functional PANoptosome complex. Collectively, these results indicate that HG, not hyperosmolarity, triggers a comprehensive form of cell death involving apoptosis, pyroptosis, and necroptosis, a phenomenon known as PANoptosis, in hRMECs, which is mediated by the formation of the PANoptosome complex.

### 3.2. HG Upregulates CD36 Expression and Activates the NOTCH/MAML Signaling Pathway

**Figure 3:**
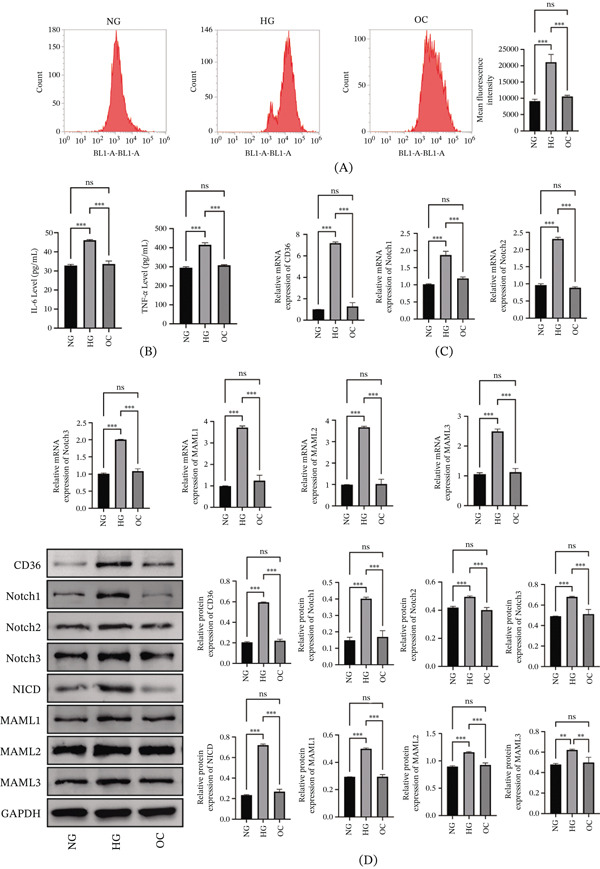
HG activates the CD36‐NOTCH/MAML pathway and induces oxidative stress and inflammation. (A) Flow cytometry analysis of ROS levels with quantification. (B) Levels of IL‐6 and TNF‐*α* in the cell supernatant detected by ELISA. (C) qPCR analysis of *CD36*, *Notch1/2/3*, and *MAML1/2/3* mRNA levels. (D) Western blot analysis of CD36, Notch1/2/3, NICD, and MAML1/2/3 protein levels with quantification. Data are presented as mean ± SD (*n* = 3). Statistical analysis was performed using one‐way ANOVA followed by Tukey′s post hoc test.  ^∗∗^ *p* < 0.01,  ^∗∗∗^ *p* < 0.001, ns = not significant.

Given that HG induced PANoptosis and PANoptosome formation, we next explored the underlying molecular mechanisms. We first assessed the expression of the scavenger receptor CD36. IF staining revealed a pronounced increase in CD36 expression and membrane localization in the HG group versus the NG and OC controls (Figure 2A, *p* < 0.001). This finding was further validated at the transcriptional and translational levels via qPCR and Western blot, respectively (Figure 3C,D; *p* < 0.001). Given the pivotal role of NOTCH/MAML signaling in endothelial cell biology, its activation status was investigated. NOTCH/MAML signaling was activated in the HG group, as evidenced by the significant upregulation of its core components (Notch1/2/3 and MAML1/2/3) at both the mRNA and protein levels (Figure 3C,D; *p* < 0.001). Activation of the NOTCH pathway, marked by the cleavage of Notch to produce the NICD, was also significantly increased in the HG group (Figure 3D, *p* < 0.001). IF staining revealed enhanced nuclear translocation and colocalization of NICD and MAML1 in hRMECs under HG conditions (Figure 2A, *p* < 0.001). Additionally, we observed elevated levels of inflammatory cytokines IL‐6 and TNF‐*α* (Figure 3B, *p* < 0.001) and increased ROS production (Figure 3A, *p* < 0.001) in the HG group. These findings suggest that HG stress leads to the upregulation of CD36 and subsequent activation of the NOTCH/MAML signaling cascade, accompanied by enhanced oxidative stress and inflammatory responses.

### 3.3. CD36 Regulates HG‐Induced PANoptosis

**Figure 4:**
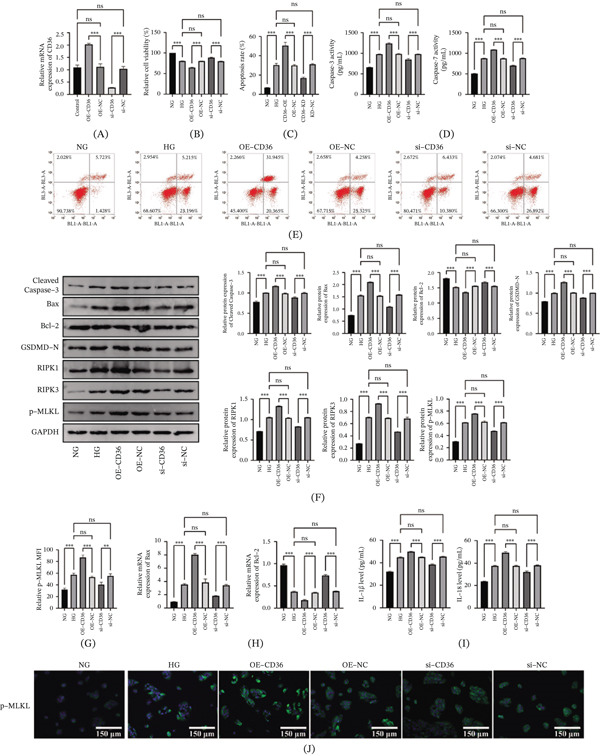
CD36 overexpression aggravates HG‐induced PANoptosis. (A) qPCR validation of CD36 transfection efficiency showing CD36 mRNA levels. (B) Cell viability measured by CCK‐8 assay. (C, E) Assessment of apoptosis by Annexin V‐FITC/PI flow cytometry. (D) Caspase‐3 and Caspase‐7 activities measured by colorimetric assay. (F) Western blot analysis of PANoptosis‐related proteins (cleaved Caspase‐3, Bax, Bcl‐2, GSDMD‐N, RIPK1, RIPK3, and p‐MLKL) with quantification. (G, J) Representative IF images and quantification showing p‐MLKL (green). DAPI (blue) was used for nuclear staining. Scale bar = 150 *μ*m. (H) qPCR analysis of *Bax* and *Bcl-2* mRNA levels. (I) ELISA detection of IL‐1*β* and IL‐18 levels in cell supernatants. Data are presented as mean ± SD (*n* = 3). Statistical analysis was performed using one‐way ANOVA followed by Tukey′s post hoc test.  ^∗∗^ *p* < 0.01,  ^∗∗∗^ *p* < 0.001, ns = not significant.

To define CD36′s role in HG‐induced PANoptosis, we modulated its expression in hRMECs via plasmid overexpression (OE‐CD36) or siRNA knockdown (si‐CD36), alongside their controls (OE‐NC and si‐NC). We also included the NG and HG groups for comparison. First, we verified the transfection efficiency by measuring CD36 mRNA levels. As shown in Figure 4A, qPCR analysis confirmed efficient CD36 modulation, with mRNA levels significantly elevated in the OE‐CD36 group and reduced in the si‐CD36 group versus their respective controls (*p* < 0.001). At the cellular functional level, CD36 overexpression significantly reduced cell viability (Figure 4B, *p* < 0.001) and markedly increased the apoptosis rate (Figure 4C,E; *p* < 0.001) compared with the HG and OE‐NC groups. Consistent with the flow cytometry data, TUNEL staining demonstrated a significantly higher proportion of TUNEL‐positive nuclei in the OE‐CD36 group and a reduced proportion in the si‐CD36 group (Supporting Information 1: Figure S1, *p* < 0.01). Conversely, CD36 knockdown rescued cell viability and attenuated the apoptosis rate induced by HG, compared with the HG and si‐NC groups (*p* < 0.001). The activity of executioner Caspase‐3/7 was significantly altered by CD36 modulation, increasing with overexpression and decreasing with knockdown (Figure 4D, *p* < 0.001).

At the molecular level, we examined the specific markers for each cell death pathway. For apoptosis, Western blot analysis (Figure 4F) revealed that OE‐CD36 significantly amplified the HG‐induced upregulation of cleaved Caspase‐3 and Bax while further decreasing the antiapoptotic protein Bcl‐2. By contrast, silencing CD36 markedly reversed these changes. This was consistent with qPCR results for *Bax* and *Bcl-2* mRNA (Figure 4H, *p* < 0.001). For pyroptosis, CD36 overexpression significantly increased the expression of GSDMD‐N, the executor of pyroptotic cell lysis (Figure 4F, *p* < 0.001). Consistently, the secretion of inflammatory cytokines IL‐1*β* and IL‐18, which are released upon pyroptotic cell death, was markedly elevated in the OE‐CD36 group (Figure 4I, *p* < 0.001). Conversely, CD36 knockdown suppressed GSDMD‐N expression and reduced IL‐1*β* and IL‐18 production. To complete the validation of the pyroptotic cascade, we evaluated the expression of NLRP3, the key upstream sensor of the canonical pyroptosis pathway and a core component of the PANoptosome. Western blot analysis demonstrated that NLRP3 protein levels were significantly upregulated by HG compared with the NG group (*p* < 0.001). Importantly, CD36 knockdown substantially reduced NLRP3 expression (*p* < 0.01), indicating that NLRP3 activation is at least partially dependent on CD36 signaling (Supporting Information 2: Figure S2). For necroptosis, the expression of RIPK1, RIPK3, and p‐MLKL was further increased in the OE‐CD36 group but decreased in the si‐CD36 group (Figure 4F, *p* < 0.001). IF staining confirmed that CD36 overexpression enhanced p‐MLKL membrane translocation, a key step in necroptosis, whereas knockdown conversely reduced it (Figure 4G,J; *p* < 0.001). These results strongly establish CD36 as a critical regulator of HG‐induced PANoptosis in hRMECs, with its overexpression exacerbating and its knockdown attenuating cellular damage and inflammatory responses through the modulation of multiple programmed cell death pathways.

### 3.4. CD36 Mediates PANoptosis Through the NOTCH/MAML Pathway

**Figure 5:**
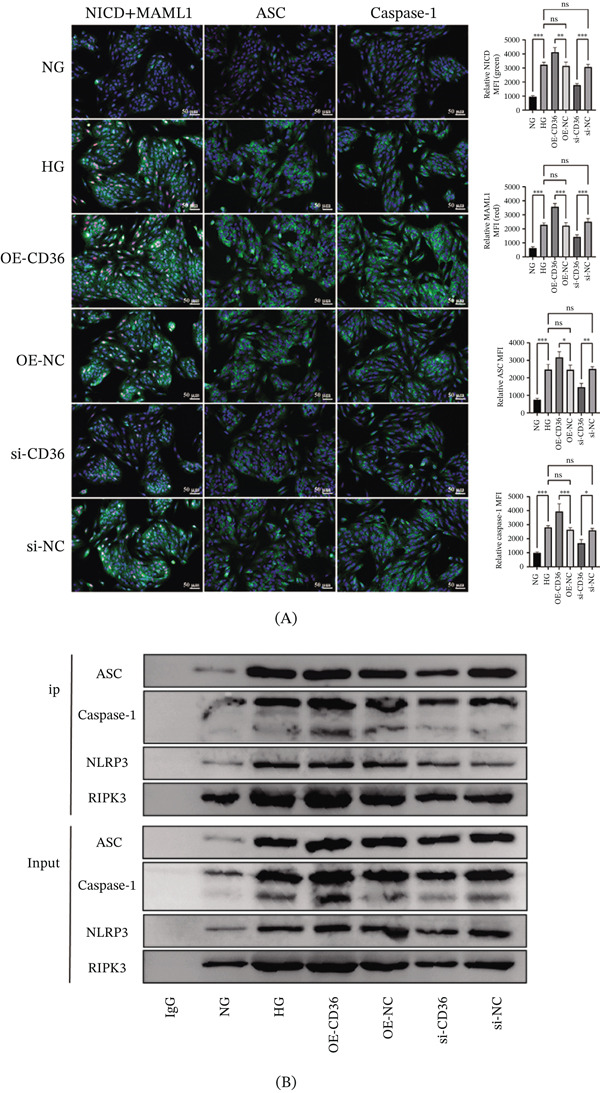
CD36 modulates PANoptosome assembly and NOTCH/MAML signaling. (A) Representative IF images and quantification showing NICD + MAML1 colocalization (green + red), ASC (green), and Caspase‐1 (green). Nuclei were counterstained with DAPI (blue). Scale bar = 50 *μ*m. (B) Co‐IP analysis showing the interaction between ASC, Caspase‐1, NLRP3, and RIPK3 in all six groups, demonstrating that CD36 overexpression enhances PANoptosome formation, whereas CD36 knockdown inhibits it. Data are presented as mean ± SD (*n* = 3). Statistical analysis was performed using one‐way ANOVA followed by Tukey′s post hoc test.  ^∗^ *p* < 0.05,  ^∗∗^ *p* < 0.01,  ^∗∗∗^ *p* < 0.001, ns = not significant.

To establish the mechanistic link between CD36 and PANoptosis, we first investigated the formation of the PANoptosome, a key multiprotein complex that executes PANoptosis by integrating apoptotic, pyroptotic, and necroptotic signaling. IF analysis showed that CD36 overexpression enhanced the formation of ASC and Caspase‐1 specks (PANoptosome components), whereas knockdown conversely suppressed it (Figure 5A, *p* < 0.05). Co‐IP experiments further confirmed that the physical interaction among PANoptosome components (ASC, Caspase‐1, NLRP3, and RIPK3) was significantly enhanced in the OE‐CD36 group and substantially weakened in the si‐CD36 group (Figure 5B), indicating that CD36 directly regulates PANoptosome assembly.

**Figure 6:**
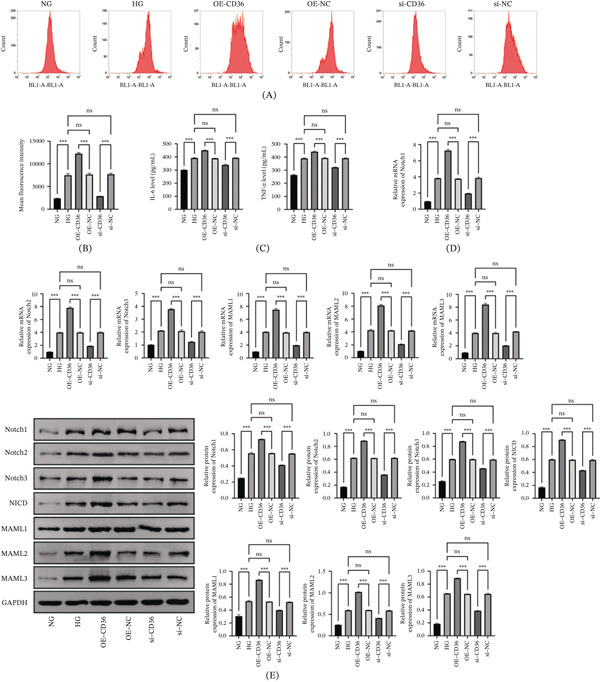
CD36 regulates the expression of NOTCH/MAML pathway components and inflammatory responses. (A) ROS levels were detected by flow cytometry. (B) Quantification of mean fluorescence intensity of ROS. (C) ELISA for IL‐6 and TNF‐*α* levels. (D) qPCR for Notch1 and MAML1 mRNA levels. (E) Western blot analysis of Notch1/2/3, NICD, and MAML1/2/3 protein levels in OE‐CD36 and si‐CD36 groups with quantification. Data are presented as mean ± SD (*n* = 3). Statistical analysis was performed using one‐way ANOVA followed by Tukey′s post hoc test.  ^∗∗∗^ *p* < 0.001, ns = not significant.

Additionally, we observed that CD36 manipulation profoundly affected cellular oxidative stress and inflammatory responses. CD36 overexpression potentiated ROS production, whereas its knockdown suppressed it (Figure 6A,B; *p* < 0.001). Similarly, CD36 overexpression upregulated the secretion of IL‐6 and TNF‐*α*, whereas knockdown downregulated these cytokines (Figure 6C, *p* < 0.001). To elucidate the upstream signaling mechanism, we examined the activation status of the NOTCH/MAML pathway following CD36 manipulation. IF staining demonstrated that CD36 overexpression promoted the nuclear colocalization of NICD and MAML1, whereas CD36 knockdown had the opposite effect (Figure 5A, *p* < 0.001). As shown by Western blot (Figure 6E), overexpression of CD36 significantly enhanced the HG‐induced upregulation of Notch1/2/3, NICD, and MAML1/2/3 proteins, whereas knockdown of CD36 substantially inhibited their expression. This was corroborated by qPCR analysis showing similar changes in Notch1 and MAML1 mRNA levels (Figure 6D, *p* < 0.001). These results indicate that CD36 acts through the NOTCH/MAML pathway to promote the assembly of the PANoptosome complex, thereby driving PANoptosis in retinal endothelial cells under hyperglycemic conditions.

### 3.5. Inhibition of the NOTCH Pathway Ameliorates HG‐Induced PANoptosis

**Figure 7:**
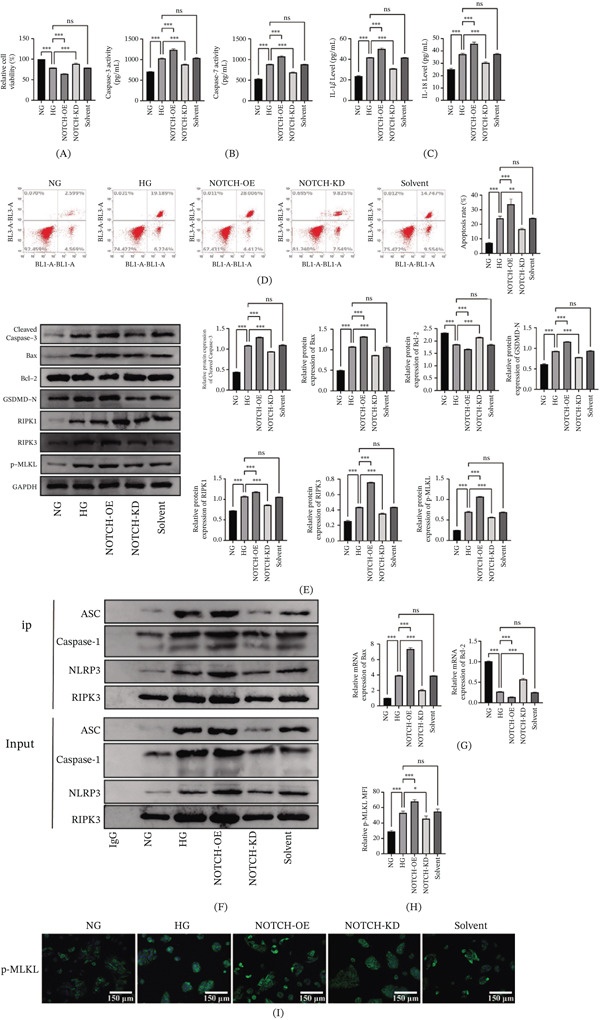
NOTCH pathway modulation affects HG‐induced PANoptosis. hRMECs were treated with a NOTCH agonist (Jagged1 peptide) or inhibitor (DAPT) under HG conditions. (A) Cell viability measured by CCK‐8 assay. (B) Caspase‐3/7 activity measured by colorimetric assay. (C) IL‐1*β* and IL‐18 levels were quantified by ELISA. (D) Apoptosis rate measured by flow cytometry with quantification. (E) Quantification of Western blot analysis for PANoptosis‐related proteins, including cleaved Caspase‐3, GSDMD‐N, RIPK1, Bax, RIPK3, p‐MLKL, and Bcl‐2. (F) Co‐IP analysis of PANoptosome components (ASC, Caspase‐1, NLRP3, and RIPK3). (G) qPCR analysis of *Bax* and *Bcl-2* mRNA levels. (H, I) IF staining of p‐MLKL (green) with DAPI (blue) and quantification. Scale bar = 150 *μ*m. Data are presented as mean ± SD (*n* = 3). Statistical analysis was performed using one‐way ANOVA followed by Tukey′s post hoc test.  ^∗^ *p* < 0.01,  ^∗∗^ *p* < 0.01,  ^∗∗∗^ *p* < 0.001, ns = not significant.

**Figure 8:**
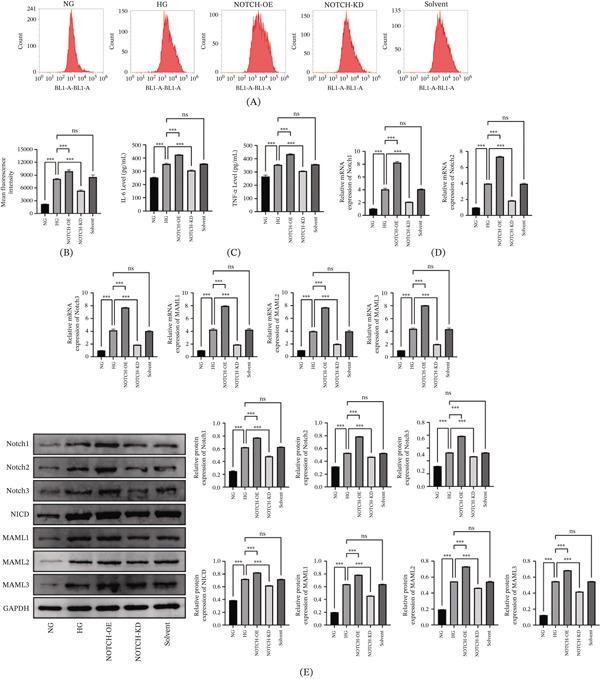
Validation of NOTCH pathway modulation on cellular responses. hRMECs were treated with NOTCH agonist or inhibitor under high glucose. (A) ROS levels were detected by flow cytometry. (B) Measurement of mean fluorescence intensity for intracellular ROS. (C) ELISA for IL‐6 and TNF‐*α* levels. (D) qPCR analysis of Notch1, Notch2, Notch3, MAML1, MAML2, and MAML3 mRNA levels. (E) Western blot analysis of Notch1/2/3, NICD, and MAML1/2/3 protein levels with quantification. Data are presented as mean ± SD (*n* = 3). Statistical analysis was performed using one‐way ANOVA followed by Tukey′s post hoc test.  ^∗∗∗^ *p* < 0.001, ns = not significant.

To definitively establish the essential role of NOTCH/MAML signaling in HG‐induced PANoptosis, we treated hRMECs with a NOTCH agonist (Jagged1 peptide and NOTCH‐OE) or inhibitor (DAPT and NOTCH‐KD) under HG conditions. At the cellular functional level, treatment with the NOTCH agonist further decreased cell viability (Figure 7A, *p* < 0.001) and increased the apoptosis rate (Figure 7D, *p* < 0.001), mimicking the effects of CD36 overexpression. NOTCH agonist treatment further augmented Caspase‐3/7 activity (Figure 7B, *p* < 0.001). Moreover, NOTCH activation led to increased secretion of inflammatory cytokines IL‐1*β*, IL‐18, IL‐6, and TNF‐*α* (Figures 7C and 8C, *p* < 0.001), as well as elevated ROS production (Figure 8A,B; *p* < 0.001). In contrast, the NOTCH inhibitor DAPT significantly alleviated HG‐induced cellular damage, improving cell viability and reducing the apoptosis rate, caspase activities, inflammatory cytokine production, and ROS levels (*p* < 0.001). At the molecular level, Western blot analysis (Figure 7E) showed that DAPT treatment effectively suppressed the HG‐induced upregulation of cleaved Caspase‐3, Bax, GSDMD‐N, RIPK1, RIPK3, and p‐MLKL while restoring Bcl‐2 expression. Additionally, qPCR analysis confirmed that NOTCH‐OE significantly upregulated Bax mRNA while downregulating Bcl‐2 mRNA, with NOTCH‐KD showing the opposite trend (Figure 7G, *p* < 0.001). Furthermore, DAPT inhibited the formation of the PANoptosome complex, as evidenced by Co‐IP showing reduced interaction among ASC, Caspase‐1, NLRP3, and RIPK3 (Figure 7F), and reduced p‐MLKL membrane translocation (Figure 7H,I). As expected, DAPT treatment also downregulated the expression of NOTCH pathway components, including Notch receptors, NICD, and MAML proteins (Figure 8D,E). Conversely, the NOTCH agonist enhanced the expression of these proteins and exacerbated PANoptosis. These results confirm that the NOTCH/MAML pathway is a critical downstream effector in the CD36‐mediated PANoptosis of retinal endothelial cells under hyperglycemic conditions, and its inhibition represents a potential therapeutic strategy for DR.

## 4. Discussion

DR is a major contributor to global vision loss, and its pathogenesis is intricately linked to hyperglycemia‐induced damage to retinal microvascular endothelial cells [24, 25]. Retinal endothelial cells are particularly vulnerable to metabolic disturbances in diabetes, as they form the critical blood–retinal barrier and maintain vascular homeostasis [26, 27]. Although multiple modalities of programmed cell death have been linked to DR, our study provides novel insights by demonstrating for the first time that HG induces a comprehensive and inflammatory form of cell death, known as PANoptosis, in hRMECs. Crucially, we have elucidated a specific molecular cascade driving this process, identifying the scavenger receptor CD36 as a key upstream regulator that activates the NOTCH/MAML signaling pathway, leading to the assembly of the PANoptosome and subsequent cell demise.

Our initial findings confirmed that HG, rather than hyperosmolarity, specifically triggers PANoptosis, characterized by the concurrent activation of apoptosis, pyroptosis, and necroptosis. This is consistent with emerging evidence highlighting the importance of PANoptosis in various inflammatory and metabolic diseases [10, 28, 29]. Importantly, recent studies have demonstrated the presence of PANoptosis‐like cell death in ischemia/reperfusion injury of retinal neurons [12], as well as in cerebral ischemia rodent models [13], underscoring its broad pathological relevance in neural and vascular tissues. Whereas previous studies have individually linked apoptosis, pyroptosis, or necroptosis to DR pathogenesis [30], our work integrates these pathways, suggesting that a coordinated PANoptotic response contributes significantly to the retinal endothelial cell loss and inflammation seen in DR. This integrated view aligns with recent bioinformatic analyses that have identified PANoptosis‐related gene signatures in DR, underscoring its clinical relevance [18]. The observed increase in oxidative stress and inflammatory cytokines in our model further supports the notion that PANoptosis is a key driver of the inflammatory microenvironment in the diabetic retina [31, 32]. Furthermore, our TUNEL morphological data corroborated the biochemical markers, firmly establishing the execution of cell death programs in our model.

A key finding of this study is that CD36 acts as a critical driver of the PANoptotic cascade. We observed a significant upregulation of CD36 expression in hRMECs exposed to HG, a finding that corroborates previous reports on the role of CD36 in diabetic complications [17]. CD36 is a multifunctional receptor known to mediate the uptake of fatty acids and oxidized lipoproteins, leading to lipotoxicity, oxidative stress, and inflammation—all of which are hallmark features of DR [33, 34]. The metabolic dysregulation in diabetes, characterized by altered glucose and lipid metabolism, profoundly affects endothelial cell function through multiple mechanisms [35, 36]. By demonstrating that overexpression of CD36 exacerbates HG‐induced PANoptosis while its knockdown provides protection, our study functionally establishes CD36 as a prodeath signal in the context of DR. This positions CD36 as a potential therapeutic target to mitigate retinal endothelial cell damage.

Furthermore, we have uncovered a novel signaling axis downstream of CD36, involving the NOTCH/MAML pathway. The highly conserved NOTCH signaling pathway orchestrates key vascular processes, including development, homeostasis, and pathogenesis [37, 38]. Its role in DR has been debated, with some studies reporting activation and others reporting inhibition [22, 39]. Our results clearly demonstrate that in hRMECs, HG‐induced CD36 expression leads to the robust activation of the NOTCH pathway, evidenced by increased expression of Notch receptors, their coactivators MAML1/2/3, and the generation of the active NICD fragment. We further showed that this activation is essential for PANoptosis, as a NOTCH agonist mimicked the detrimental effects of CD36 overexpression, whereas a NOTCH inhibitor (DAPT) rescued the cells. Our findings establish the CD36‐NOTCH axis as a pivotal regulator of endothelial cell fate under hyperglycemic stress. Crucially, our study highlights the crosstalk between the NOTCH pathway and the NLRP3 inflammasome. Previous research has demonstrated that Notch1 signaling can regulate NLRP3 inflammasome function [40], and CD36 has been identified as a central coordinator of NLRP3 inflammasome activation [19]. In our model, we observed that CD36 knockdown significantly reduced NLRP3 expression, suggesting that the CD36‐NOTCH axis mediates PANoptosis at least in part through the transcriptional or posttranslational regulation of NLRP3. This positions NLRP3 as a critical downstream effector linking NOTCH signaling to the execution of pyroptosis within the PANoptotic cascade. The activation of this pathway appears to be a key step in translating the stress signal from CD36 into the assembly of the PANoptosome, the central executioner complex of PANoptosis [28, 29].

In conclusion, our study delineates a previously uncharacterized pathway in the pathogenesis of DR: HG upregulates CD36, which in turn activates the NOTCH/MAML signaling cascade, culminating in NLRP3‐dependent PANoptosome assembly and the execution of PANoptosis in retinal endothelial cells. This cascade of events contributes to the vascular cell loss and inflammation that drive the progression of DR. While this study provides a comprehensive in vitro analysis, further validation in in vivo animal models of DR is warranted to confirm these findings. Future research should also aim to identify the specific CD36 ligands involved and explore the potential crosstalk with other signaling pathways. Nevertheless, our findings highlight the CD36‐NOTCH‐NLRP3‐PANoptosis axis, offering a promising novel strategy for treating DR and a potential avenue to prevent or slow the progression of this devastating disease.

**Nomenclature**
ANOVA: analysis of variance
ASC: apoptosis‐associated speck‐like protein containing a CARD
Bax: Bcl‐2‐associated X protein
BCA: bicinchoninic acid
Bcl‐2: B‐cell Lymphoma 2
BSA: bovine serum albumin
CCK‐8: Cell Counting Kit‐8
Co‐IP: coimmunoprecipitation
DAPI: 4 ^′^,6‐diamidino‐2‐phenylindole
DAPT: *N*‐[*N*‐(3,5‐difluorophenacetyl)‐L‐alanyl]‐S‐phenylglycine t‐butyl ester
DCFH‐DA: 2 ^′^,7 ^′^‐dichlorodihydrofluorescein diacetate
DMSO: dimethyl sulfoxide
DR: diabetic retinopathy
ELISA: enzyme‐linked immunosorbent assay
FITC: fluorescein isothiocyanate
GAPDH: glyceraldehyde‐3‐phosphate dehydrogenase
GSDMD‐N: Gasdermin D N‐terminal fragment
HG: high glucose
hRMECs: human retinal microvascular endothelial cells
HRP: horseradish peroxidase
IF: immunofluorescence
IgG: Immunoglobulin G
IL‐1*β*/6/18: Interleukin‐1 beta/6/18
MAML: Mastermind‐like
NC: negative control
NG: normal glucose
NICD: Notch intracellular domain
NLRP3: NLR Family Pyrin Domain‐Containing 3
OC: osmotic control
OE: overexpression
PBS: phosphate‐buffered saline
PI: propidium iodide
p‐MLKL: phosphorylated mixed lineage kinase domain‐like pseudokinase
PVDF: polyvinylidene fluoride
qPCR: quantitative polymerase chain reaction
RIPA: radioimmunoprecipitation assay
RIPK1/3: receptor‐interacting serine/threonine‐protein Kinase 1/3
ROS: reactive oxygen species
RPE: retinal pigment epithelium
SD: standard deviation
SDS‐PAGE: sodium dodecyl sulfate polyacrylamide gel electrophoresis
siRNA: small interfering RNA
TNF‐*α*
: tumor necrosis factor‐alpha
VEGF: vascular endothelial growth factor

## Author Contributions

Rongfeng Dai: conceptualization, methodology, formal analysis, investigation, supervision, and writing—review and editing; Yu Qian: investigation, data curation, and formal analysis; Siqi Liu: investigation and data curation; Yuqing You: conceptualization, investigation, resources, and writing—original draft; Yan Han: writing—original draft and writing—review and editing; Yi Fang: investigation and methodology.

## Funding

No funding was received for this manuscript.

## Ethics Statement

The authors have nothing to report.

## Consent

The authors have nothing to report.

## Conflicts of Interest

The authors declare no conflicts of interest.

## Supporting Information

Additional supporting information can be found online in the Supporting Information section.

## Supporting information


**Supporting Information 1** Figure S1: TUNEL staining for morphological validation of cell death. Representative fluorescence microscopy images of TUNEL staining (red), DAPI nuclear staining (blue), and merged images in NG, HG, OE‐CD36, OE‐NC, si‐CD36, and si‐NC groups. Quantification of the apoptosis rate (percentage of TUNEL‐positive cells) is shown in the bar graph. Scale bar = 50 *μ*m. Data are presented as mean ± SD (*n* = 3). Statistical analysis was performed using one‐way ANOVA followed by Tukey′s post hoc test.  ^∗∗^ *p* < 0.01,  ^∗∗∗^ *p* < 0.001, ns = not significant.

**Supporting Information 2** Figure S2: NLRP3 protein expression detected by Western blotting. Representative Western blot images and quantification of NLRP3 protein levels in NG, HG, OE‐CD36, OE‐NC, si‐CD36, and si‐NC groups. GAPDH served as the loading control. Data are presented as mean ± SD (*n* = 3). Statistical analysis was performed using one‐way ANOVA followed by Tukey′s post hoc test.  ^∗∗^ *p* < 0.01,  ^∗∗∗^ *p* < 0.001, ns = not significant.

**Supporting Information 3** Mechanistic diagram: schematic diagram of the CD36‐NOTCH‐NLRP3‐PANoptosis axis in hRMECs under HG conditions. HG upregulates the expression of the scavenger receptor CD36, which in turn activates the NOTCH/MAML signaling pathway, characterized by increased *γ*‐secretase‐mediated cleavage to produce NICD and its subsequent nuclear colocalization with MAML1. This activation promotes the transcription and expression of NLRP3. The elevated NLRP3, along with ASC, Caspase‐1, and PIPK3, assembles into the PANoptosome complex. This multiprotein complex concurrently drives apoptosis (via cleaved Caspase‐3 and Bax upregulation/Bcl‐2 downregulation), pyroptosis (via GSDMD cleavage and IL‐18/IL‐1*β* release), and necroptosis (via PIPK1/PIPK3‐mediated MLKL phosphorylation), ultimately culminating in PANoptosis. Pharmacological inhibition of NOTCH by DAPT effectively blocks this signaling cascade, mitigating HG‐induced cell death.

**Supporting Information 4** Table S1: Primer sequences used in the qRT‐PCR experiments. Table S2: Antibodies used in Western blot analyses. Table S3: Antibodies used in IF and Co‐IP analyses.

**Supporting Information 5** Figure S3: Uncropped Western blot membranes corresponding to Figure 1E of the manuscript. Molecular weight markers were added, and the expected positions of the proteins were indicated. The red box indicates the region used in the final figure. Figure S4: Uncropped Western blot membranes corresponding to Figure 3D of the manuscript. Molecular weight markers were added, and the expected positions of the proteins were indicated. The red box indicates the region used in the final figure. Figure S5: Uncropped Western blot membranes corresponding to Figure 4F of the manuscript. Molecular weight markers were added, and the expected positions of the proteins were indicated. The red box indicates the region used in the final figure. Figure S6: Uncropped Western blot membranes corresponding to Figure 6E of the manuscript. Molecular weight markers were added, and the expected positions of the proteins were indicated. The red box indicates the region used in the final figure. Figure S7: Uncropped Western blot membranes corresponding to Figure 7E of the manuscript. Molecular weight markers were added, and the expected positions of the proteins were indicated. The red box indicates the region used in the final figure. Figure S8: Uncropped Western blot membranes corresponding to Figure 8E of the manuscript. Molecular weight markers were added, and the expected positions of the proteins were indicated. The red box indicates the region used in the final figure.

## Data Availability

The data that support the findings of this study are available from the corresponding author upon reasonable request.
