# Supplementary figures and images for: CD36 Regulates PANoptosis in Diabetic Retinopathy via the NOTCH/MAML Pathway
Source: J Diabetes Res. 2026 Jul 24;2026:9324498. doi: 10.1155/jdr/9324498 (PMC13397476; doi:10.1155/jdr/9324498)

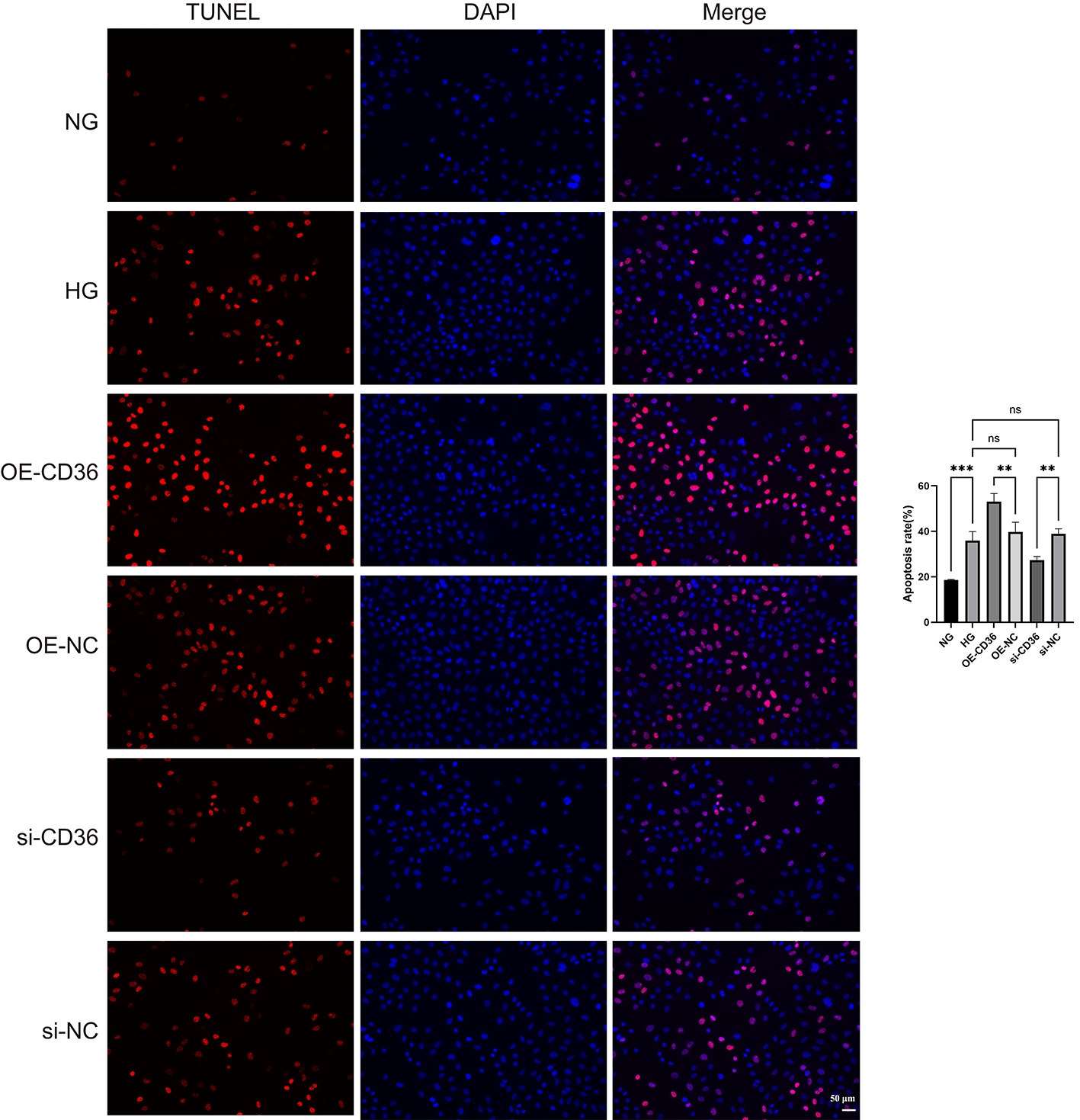

Supplement: Supplementary file 1 — Supporting Information 1 Figure S1: TUNEL staining for morphological validation of cell death. Representative fluorescence microscopy images of TUNEL staining (red), DAPI nuclear staining (blue), and merged images in NG, HG, OE‐CD36, OE‐NC, si‐CD36, and si‐NC groups. Quantification of the apoptosis rate (percentage of TUNEL‐positive cells) is shown in the bar graph. Scale bar = 50 μm. Data are presented as mean ± SD (n = 3). Statistical analysis was performed using one‐way ANOVA followed by Tukey′s post hoc test. ∗∗ p < 0.01, ∗∗∗ p < 0.001, ns = not significant. [file JDR-2026-9324498-s001.tif]

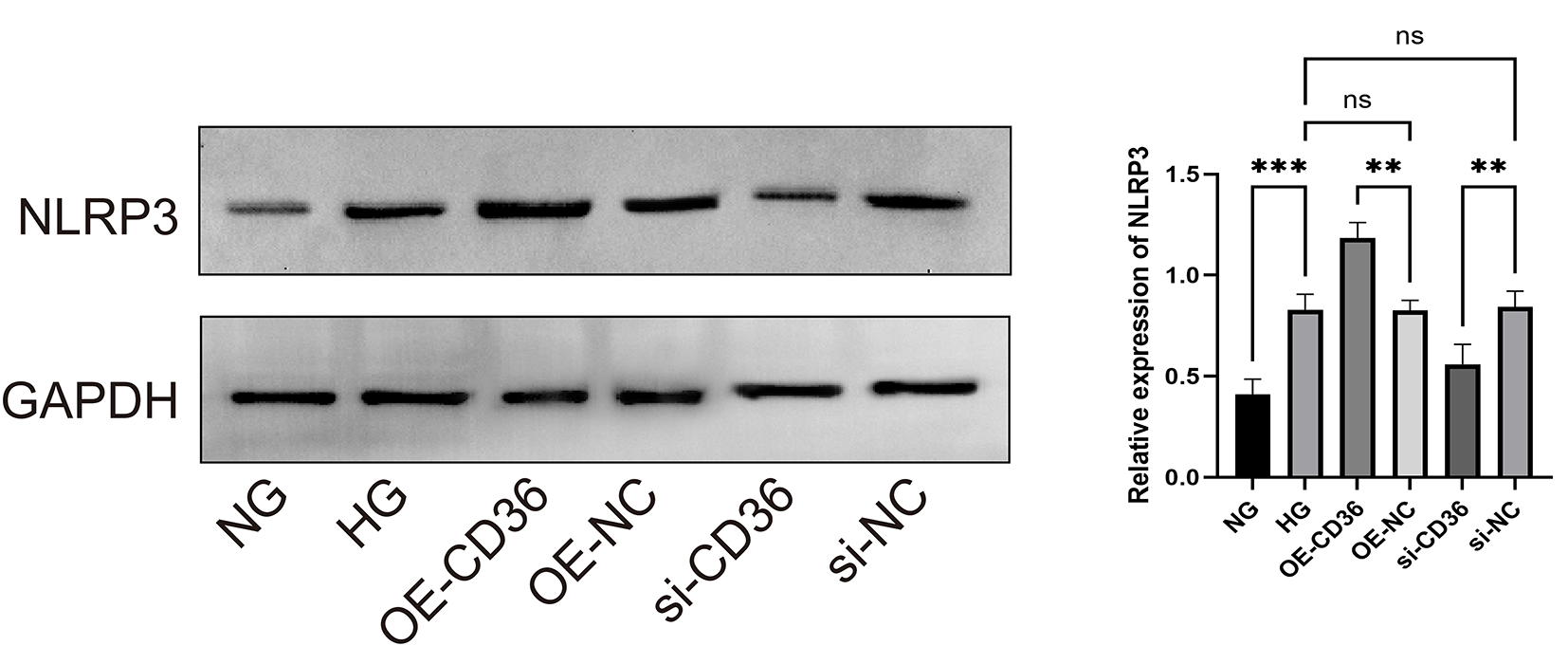

Supplement: Supplementary file 2 — Supporting Information 2 Figure S2: NLRP3 protein expression detected by Western blotting. Representative Western blot images and quantification of NLRP3 protein levels in NG, HG, OE‐CD36, OE‐NC, si‐CD36, and si‐NC groups. GAPDH served as the loading control. Data are presented as mean ± SD (n = 3). Statistical analysis was performed using one‐way ANOVA followed by Tukey′s post hoc test. ∗∗ p < 0.01, ∗∗∗ p < 0.001, ns = not significant. [file JDR-2026-9324498-s002.tif]

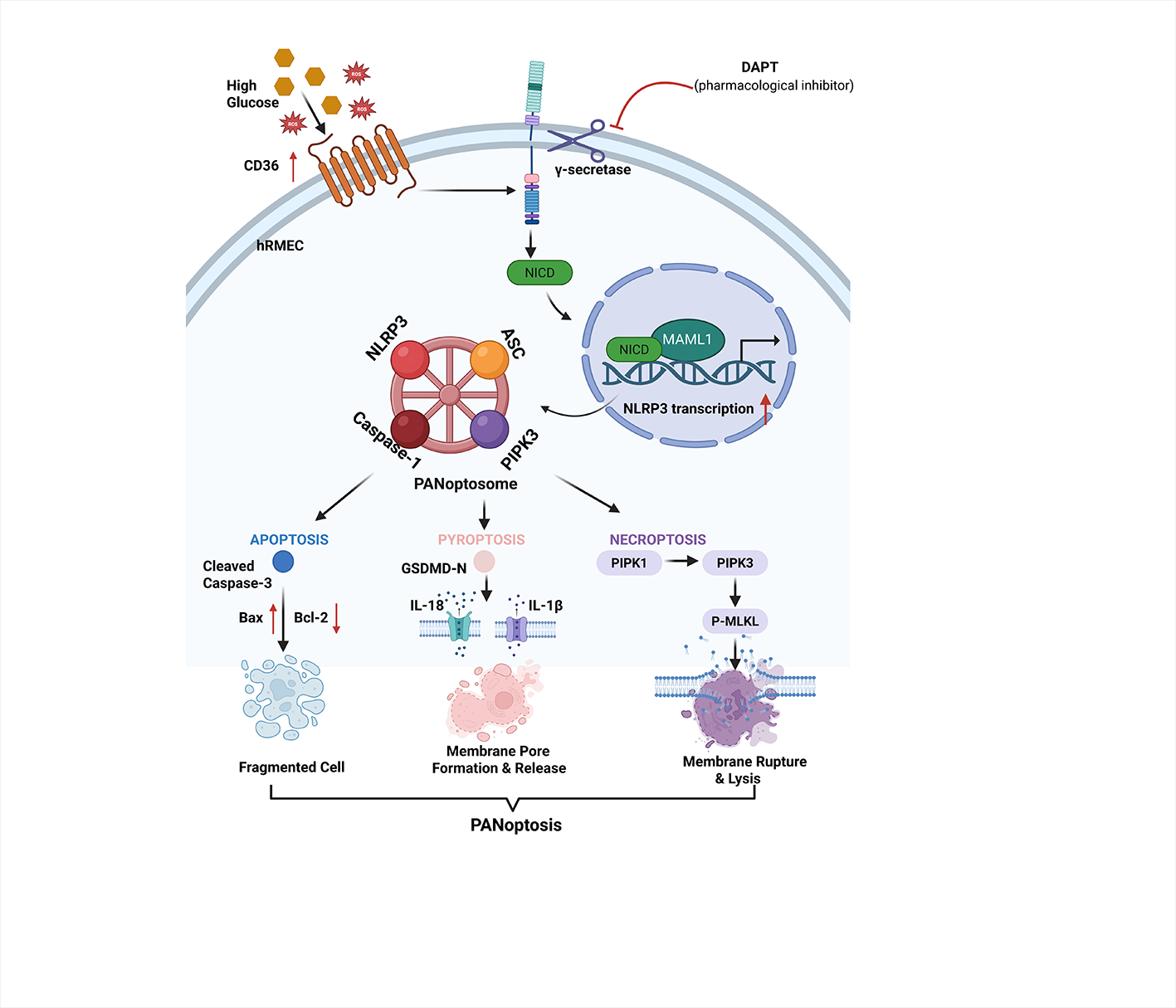

Supplement: Supplementary file 3 — Supporting Information 3 Mechanistic diagram: schematic diagram of the CD36‐NOTCH‐NLRP3‐PANoptosis axis in hRMECs under HG conditions. HG upregulates the expression of the scavenger receptor CD36, which in turn activates the NOTCH/MAML signaling pathway, characterized by increased γ‐secretase‐mediated cleavage to produce NICD and its subsequent nuclear colocalization with MAML1. This activation promotes the transcription and expression of NLRP3. The elevated NLRP3, along with ASC, Caspase‐1, and PIPK3, assembles into the PANoptosome complex. This multiprotein complex concurrently drives apoptosis (via cleaved Caspase‐3 and Bax upregulation/Bcl‐2 downregulation), pyroptosis (via GSDMD cleavage and IL‐18/IL‐1β release), and necroptosis (via PIPK1/PIPK3‐mediated MLKL phosphorylation), ultimately culminating in PANoptosis. Pharmacological inhibition of NOTCH by DAPT effectively blocks this signaling cascade, mitigating HG‐induced cell death. [file JDR-2026-9324498-s005.tif]

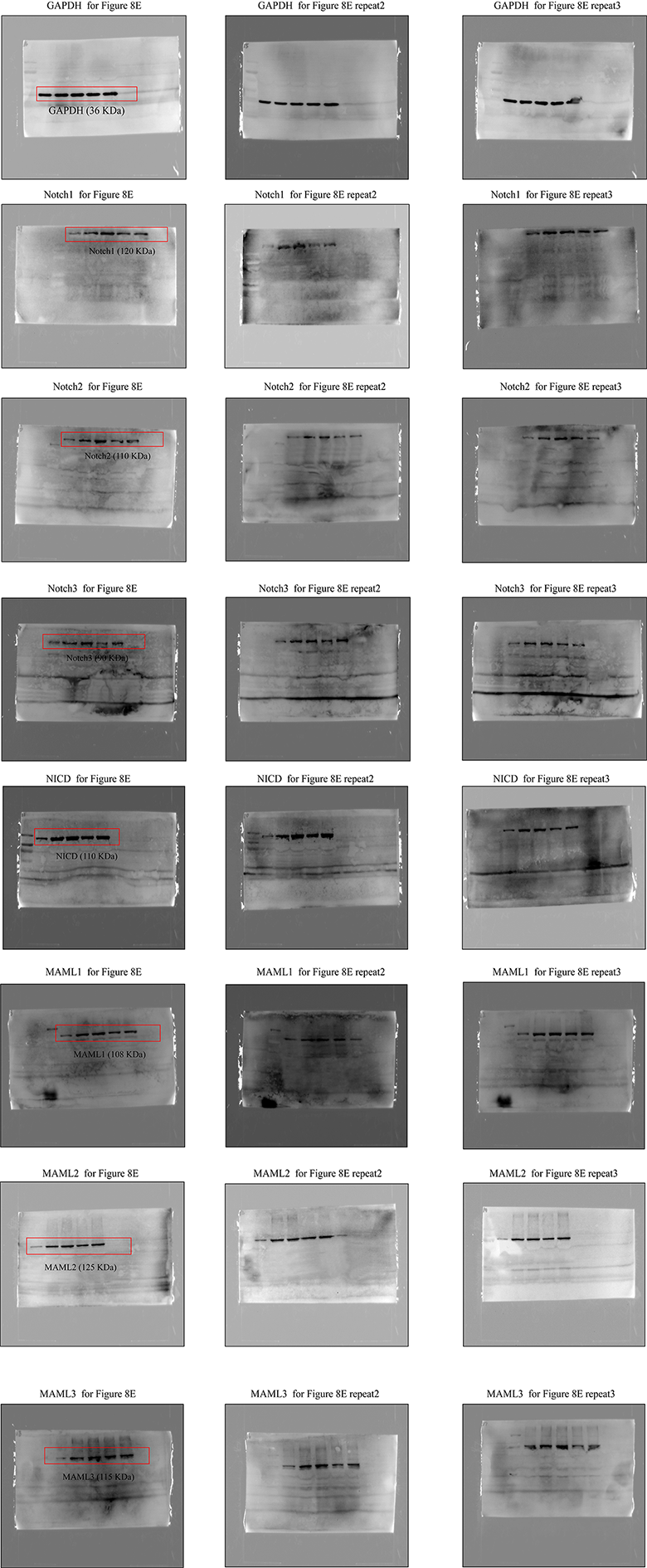

Supplement: Supplementary file 5 — Supporting Information 5 Figure S3: Uncropped Western blot membranes corresponding to Figure 1E of the manuscript. Molecular weight markers were added, and the expected positions of the proteins were indicated. The red box indicates the region used in the final figure. Figure S4: Uncropped Western blot membranes corresponding to Figure 3D of the manuscript. Molecular weight markers were added, and the expected positions of the proteins were indicated. The red box indicates the region used in the final figure. Figure S5: Uncropped Western blot membranes corresponding to Figure 4F of the manuscript. Molecular weight markers were added, and the expected positions of the proteins were indicated. The red box indicates the region used in the final figure. Figure S6: Uncropped Western blot membranes corresponding to Figure 6E of the manuscript. Molecular weight markers were added, and the expected positions of the proteins were indicated. The red box indicates the region used in the final figure. Figure S7: Uncropped Western blot membranes corresponding to Figure 7E of the manuscript. Molecular weight markers were added, and the expected positions of the proteins were indicated. The red box indicates the region used in the final figure. Figure S8: Uncropped Western blot membranes corresponding to Figure 8E of the manuscript. Molecular weight markers were added, and the expected positions of the proteins were indicated. The red box indicates the region used in the final figure. [file JDR-2026-9324498-s003.zip › Figure S8.tif]

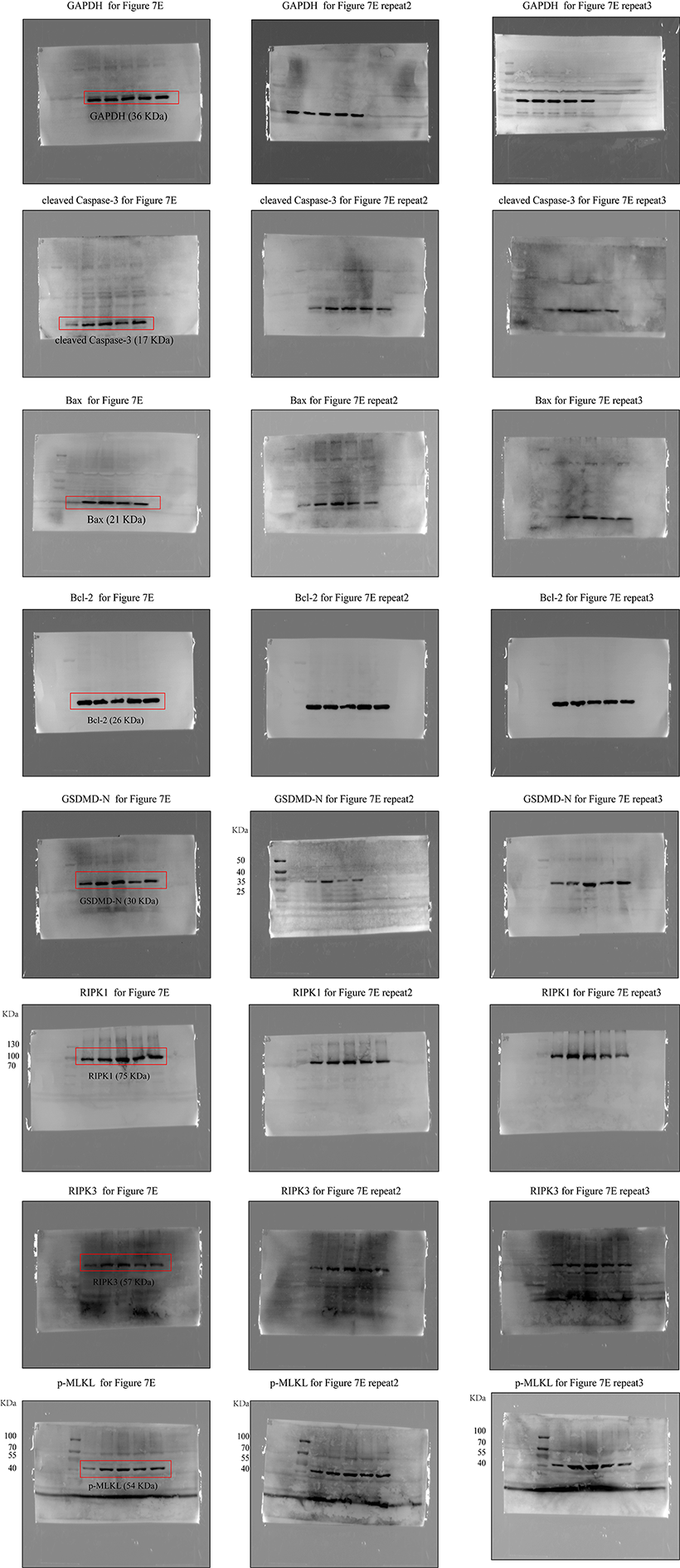

Supplement: Supplementary file 5 — Supporting Information 5 Figure S3: Uncropped Western blot membranes corresponding to Figure 1E of the manuscript. Molecular weight markers were added, and the expected positions of the proteins were indicated. The red box indicates the region used in the final figure. Figure S4: Uncropped Western blot membranes corresponding to Figure 3D of the manuscript. Molecular weight markers were added, and the expected positions of the proteins were indicated. The red box indicates the region used in the final figure. Figure S5: Uncropped Western blot membranes corresponding to Figure 4F of the manuscript. Molecular weight markers were added, and the expected positions of the proteins were indicated. The red box indicates the region used in the final figure. Figure S6: Uncropped Western blot membranes corresponding to Figure 6E of the manuscript. Molecular weight markers were added, and the expected positions of the proteins were indicated. The red box indicates the region used in the final figure. Figure S7: Uncropped Western blot membranes corresponding to Figure 7E of the manuscript. Molecular weight markers were added, and the expected positions of the proteins were indicated. The red box indicates the region used in the final figure. Figure S8: Uncropped Western blot membranes corresponding to Figure 8E of the manuscript. Molecular weight markers were added, and the expected positions of the proteins were indicated. The red box indicates the region used in the final figure. [file JDR-2026-9324498-s003.zip › Figure S7.tif]

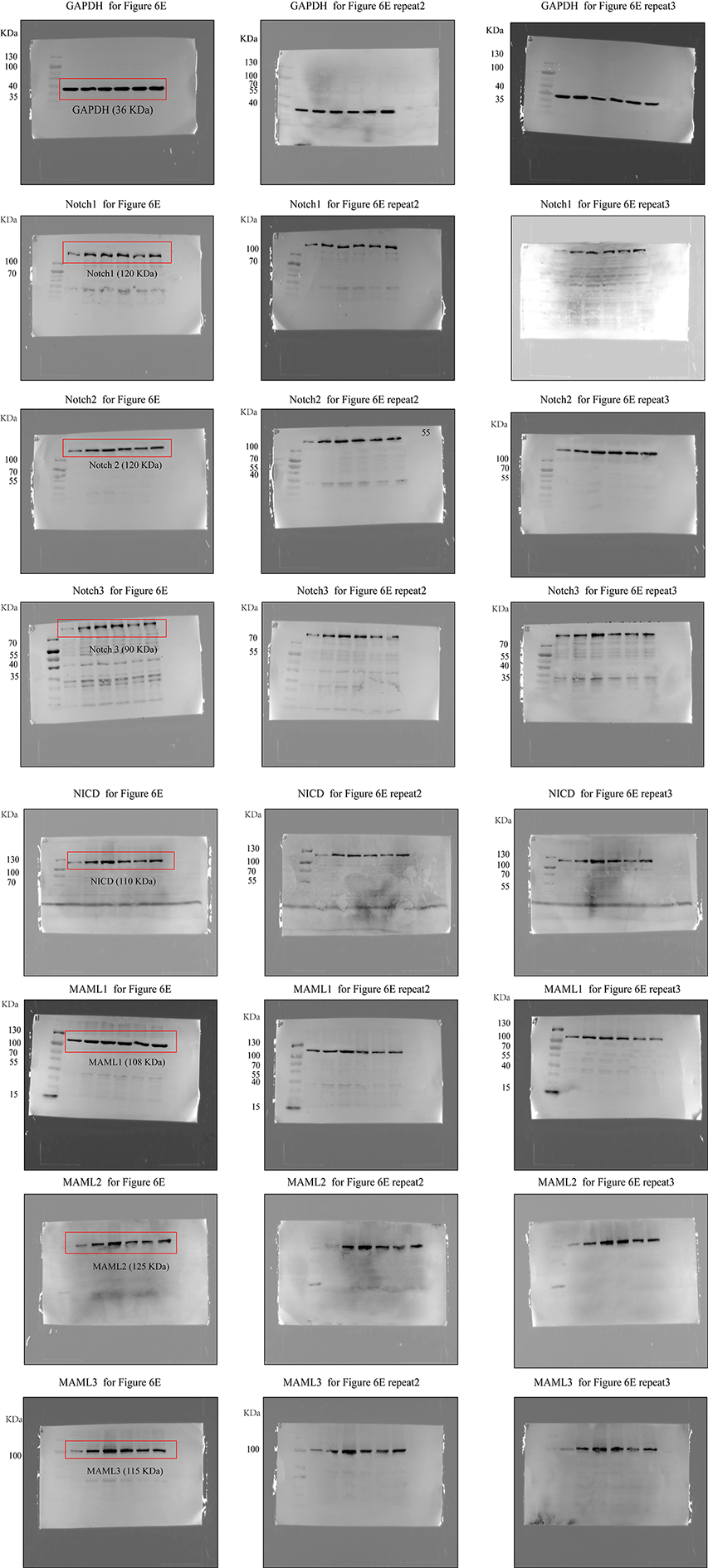

Supplement: Supplementary file 5 — Supporting Information 5 Figure S3: Uncropped Western blot membranes corresponding to Figure 1E of the manuscript. Molecular weight markers were added, and the expected positions of the proteins were indicated. The red box indicates the region used in the final figure. Figure S4: Uncropped Western blot membranes corresponding to Figure 3D of the manuscript. Molecular weight markers were added, and the expected positions of the proteins were indicated. The red box indicates the region used in the final figure. Figure S5: Uncropped Western blot membranes corresponding to Figure 4F of the manuscript. Molecular weight markers were added, and the expected positions of the proteins were indicated. The red box indicates the region used in the final figure. Figure S6: Uncropped Western blot membranes corresponding to Figure 6E of the manuscript. Molecular weight markers were added, and the expected positions of the proteins were indicated. The red box indicates the region used in the final figure. Figure S7: Uncropped Western blot membranes corresponding to Figure 7E of the manuscript. Molecular weight markers were added, and the expected positions of the proteins were indicated. The red box indicates the region used in the final figure. Figure S8: Uncropped Western blot membranes corresponding to Figure 8E of the manuscript. Molecular weight markers were added, and the expected positions of the proteins were indicated. The red box indicates the region used in the final figure. [file JDR-2026-9324498-s003.zip › Figure S6.tif]

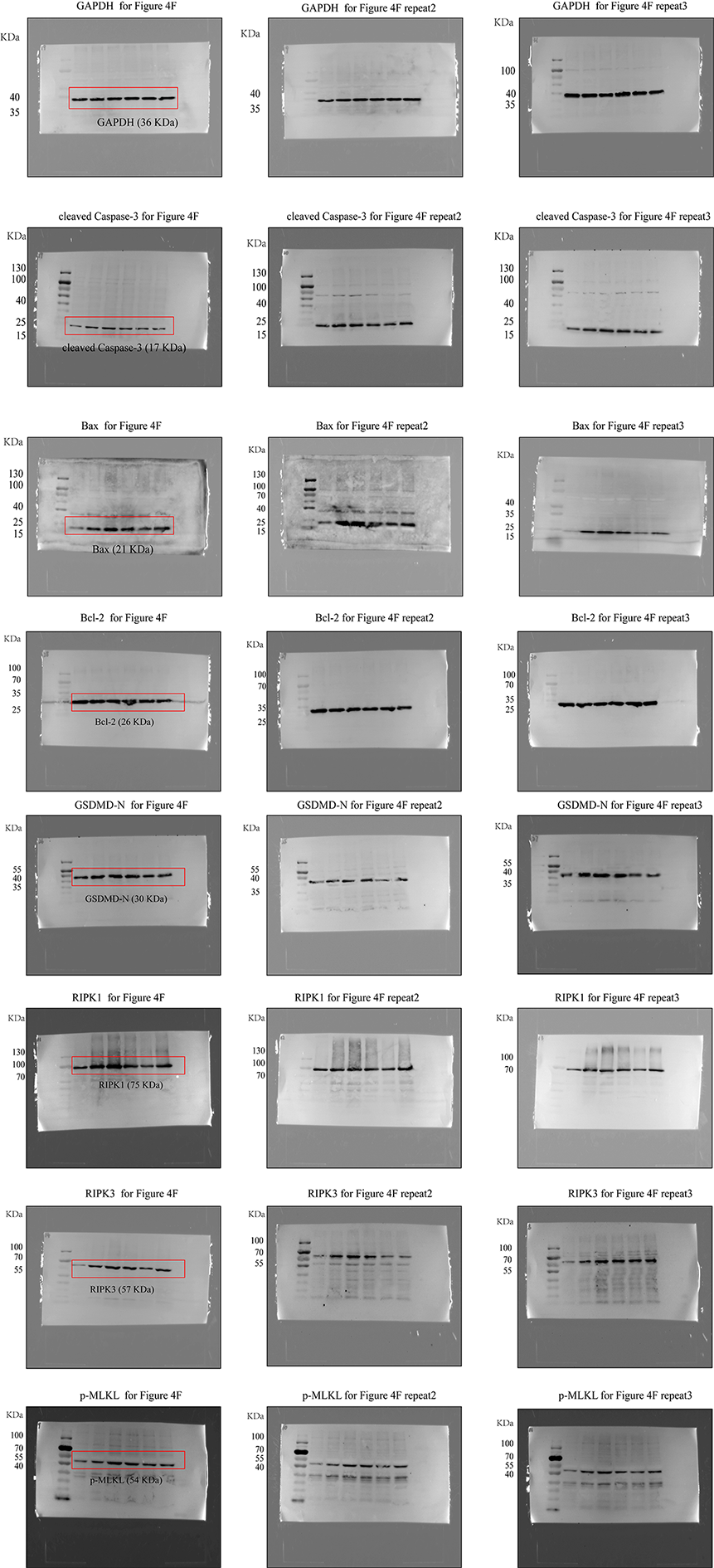

Supplement: Supplementary file 5 — Supporting Information 5 Figure S3: Uncropped Western blot membranes corresponding to Figure 1E of the manuscript. Molecular weight markers were added, and the expected positions of the proteins were indicated. The red box indicates the region used in the final figure. Figure S4: Uncropped Western blot membranes corresponding to Figure 3D of the manuscript. Molecular weight markers were added, and the expected positions of the proteins were indicated. The red box indicates the region used in the final figure. Figure S5: Uncropped Western blot membranes corresponding to Figure 4F of the manuscript. Molecular weight markers were added, and the expected positions of the proteins were indicated. The red box indicates the region used in the final figure. Figure S6: Uncropped Western blot membranes corresponding to Figure 6E of the manuscript. Molecular weight markers were added, and the expected positions of the proteins were indicated. The red box indicates the region used in the final figure. Figure S7: Uncropped Western blot membranes corresponding to Figure 7E of the manuscript. Molecular weight markers were added, and the expected positions of the proteins were indicated. The red box indicates the region used in the final figure. Figure S8: Uncropped Western blot membranes corresponding to Figure 8E of the manuscript. Molecular weight markers were added, and the expected positions of the proteins were indicated. The red box indicates the region used in the final figure. [file JDR-2026-9324498-s003.zip › Figure S5.tif]

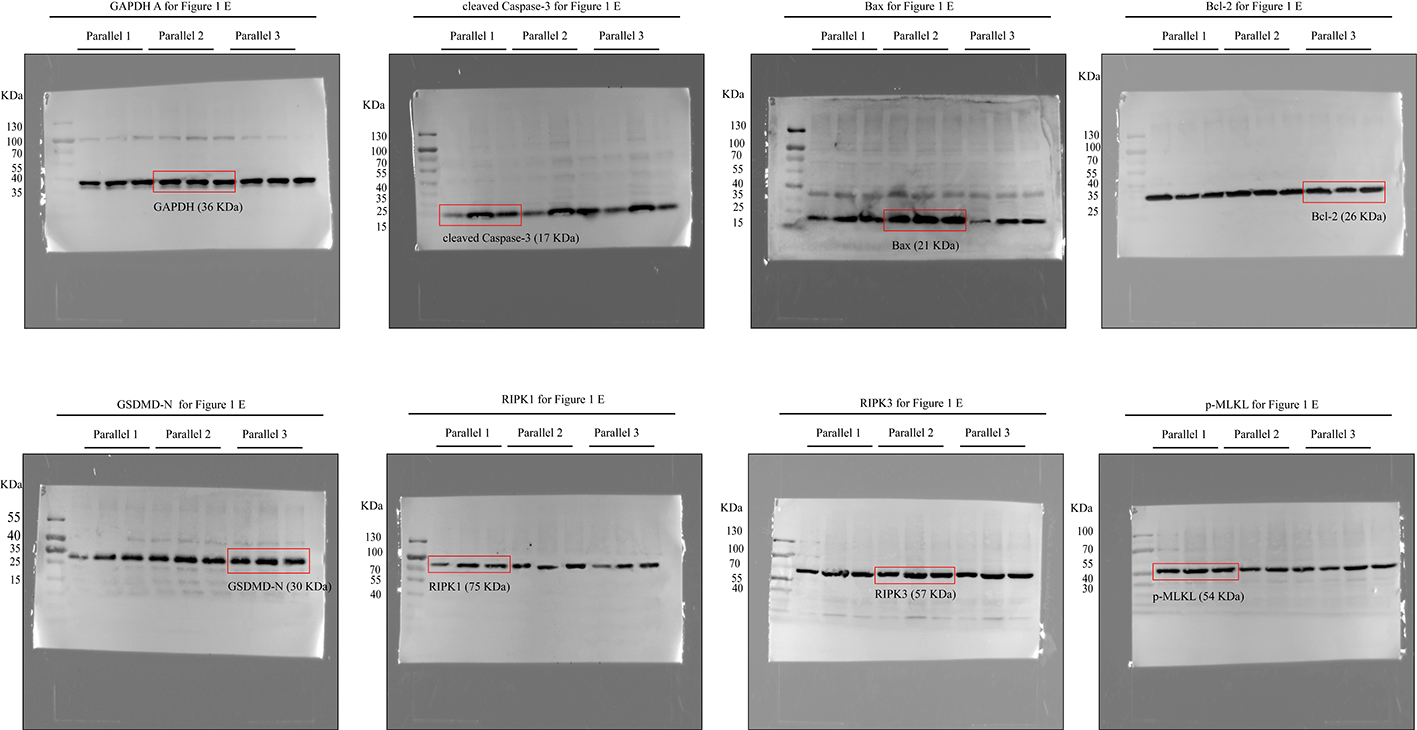

Supplement: Supplementary file 5 — Supporting Information 5 Figure S3: Uncropped Western blot membranes corresponding to Figure 1E of the manuscript. Molecular weight markers were added, and the expected positions of the proteins were indicated. The red box indicates the region used in the final figure. Figure S4: Uncropped Western blot membranes corresponding to Figure 3D of the manuscript. Molecular weight markers were added, and the expected positions of the proteins were indicated. The red box indicates the region used in the final figure. Figure S5: Uncropped Western blot membranes corresponding to Figure 4F of the manuscript. Molecular weight markers were added, and the expected positions of the proteins were indicated. The red box indicates the region used in the final figure. Figure S6: Uncropped Western blot membranes corresponding to Figure 6E of the manuscript. Molecular weight markers were added, and the expected positions of the proteins were indicated. The red box indicates the region used in the final figure. Figure S7: Uncropped Western blot membranes corresponding to Figure 7E of the manuscript. Molecular weight markers were added, and the expected positions of the proteins were indicated. The red box indicates the region used in the final figure. Figure S8: Uncropped Western blot membranes corresponding to Figure 8E of the manuscript. Molecular weight markers were added, and the expected positions of the proteins were indicated. The red box indicates the region used in the final figure. [file JDR-2026-9324498-s003.zip › Figure S3.tif]

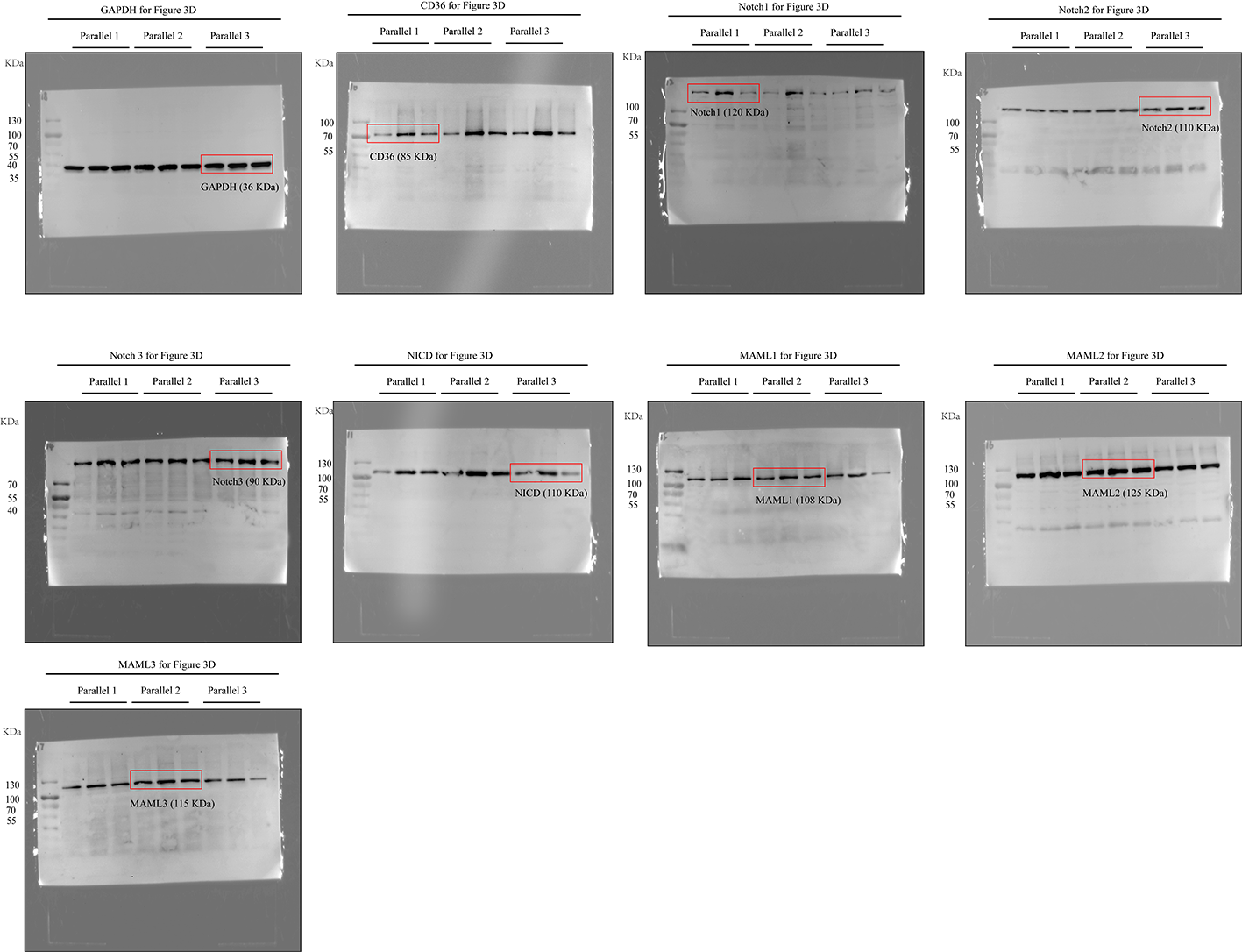

Supplement: Supplementary file 5 — Supporting Information 5 Figure S3: Uncropped Western blot membranes corresponding to Figure 1E of the manuscript. Molecular weight markers were added, and the expected positions of the proteins were indicated. The red box indicates the region used in the final figure. Figure S4: Uncropped Western blot membranes corresponding to Figure 3D of the manuscript. Molecular weight markers were added, and the expected positions of the proteins were indicated. The red box indicates the region used in the final figure. Figure S5: Uncropped Western blot membranes corresponding to Figure 4F of the manuscript. Molecular weight markers were added, and the expected positions of the proteins were indicated. The red box indicates the region used in the final figure. Figure S6: Uncropped Western blot membranes corresponding to Figure 6E of the manuscript. Molecular weight markers were added, and the expected positions of the proteins were indicated. The red box indicates the region used in the final figure. Figure S7: Uncropped Western blot membranes corresponding to Figure 7E of the manuscript. Molecular weight markers were added, and the expected positions of the proteins were indicated. The red box indicates the region used in the final figure. Figure S8: Uncropped Western blot membranes corresponding to Figure 8E of the manuscript. Molecular weight markers were added, and the expected positions of the proteins were indicated. The red box indicates the region used in the final figure. [file JDR-2026-9324498-s003.zip › Figure S4.tif]
