# Supplementary material for: CD36 Regulates PANoptosis in Diabetic Retinopathy via the NOTCH/MAML Pathway
Source: J Diabetes Res. 2026 Jul 24;2026:9324498. doi: 10.1155/jdr/9324498 (PMC13397476; doi:10.1155/jdr/9324498)
Supplement: Supplementary file 4 — Supporting Information 4 Table S1: Primer sequences used in the qRT‐PCR experiments. Table S2: Antibodies used in Western blot analyses. Table S3: Antibodies used in IF and Co‐IP analyses. [file JDR-2026-9324498-s004.docx]

**Supplemental Material**

**CD36 Promotes PANoptosis in Diabetic Retinopathy via Suppression of the NOTCH/MAML Signaling Pathway**

**Table S1**. Primer sequences used in the qRT-PCR experiments.

| Gene | Forward primer (5′–3′) | Reverse primer (3′–5′) |
| --- | --- | --- |
| Caspase-3 | AGGACTCTAGACGGCATCCA | CAGTGAGACACTCGCTCAGCTTC |
| Bax | TTCATCCAGGATCGAGCAGG | TGAGACACTCGCTCAGCTTC |
| Bcl-2 | TGGACAACCATGACCTTGGACAATCA | TCCATCCTCCACCAGTGTTCCCATC |
| CD36 | CAGGTCAACCTATTGGTCAAGCC | GCCTTCTCATCACCAATGGTCC |
| Notch1 | GGTGAACTGCTCTGAGGAGATC | GGATTGCAGTCGTCCACGTTGA |
| Notch2 | CCCAATGGGCAAGAAGTCTA | CTTGTACTCCGTCAGCGTGA |
| Notch3 | TACTGGTAGCCACTGTGAGCAG | CAGTTATCACCATTGTAGCCAGG |
| MAML1 | ACAAGTCCCAAGGGTGTCAG | TCACACAGCTGTTCCCAGAC |
| MAML2 | TTTCCCCTCAGGATCAGATG | AGAGGAGCCACCCGAATACT |
| MAML3 | CTGAAGCTGCTCAACCTCAAG | GGAGTCCTTGAGCTCCTCTTC |
| GAPDH | GAAGGTGAAGGTCGGAGTC | GAAGATGGTGATGGGATTTC |

**Table S2.** Antibodies used in western blot analyses

| **Antibody** | **Manufacturer** | **Host** | **Catalog** | **Dilution** | **Assay** |
| --- | --- | --- | --- | --- | --- |
| GAPDH | Cell Signaling Technology, USA | Rabbit | #2118 | 1:1000 | WB |
| Cleaved Caspase-3 | Cell Signaling Technology, USA | Rabbit | #9661 | 1:1000 | WB |
| Bax | Cell Signaling Technology, USA | Rabbit | #2772 | 1:1000 | WB |
| NLRP3 | Proteintech, USA | Rabbit | 19771-1-AP | 1:1000 | WB |
| Bcl-2 | Cell Signaling Technology, USA | Rabbit | #3498 | 1:1000 | WB |
| GSDMD-N | Abcam, UK | Rabbit | ab209845 | 1:1000 | WB |
| RIPK1 | Cell Signaling Technology, USA | Rabbit | #3493 | 1:1000 | WB |
| RIPK3 | Cell Signaling Technology, USA | Rabbit | #13526 | 1:1000 | WB |
| p-MLKL | Cell Signaling Technology, USA | Rabbit | #91689 | 1:1000 | WB |
| CD36 | Cell Signaling Technology, USA | Rabbit | #28109 | 1:1000 | WB |
| Notch1 | Cell Signaling Technology, USA | Rabbit | #3608 | 1:1000 | WB |
| Notch2 | Cell Signaling Technology, USA | Rabbit | #5732 | 1:1000 | WB |
| Notch3 | Cell Signaling Technology, USA | Rabbit | #2889 | 1:1000 | WB |
| NICD | Abcam, UK | Rabbit | ab8925 | 1:1000 | WB |
| MAML1 | Abcam, UK | Rabbit | ab84548 | 1:1000 | WB |
| MAML2 | Proteintech, USA | Rabbit | 16442-1-AP | 1:1000 | WB |
| MAML3 | Proteintech, USA | Rabbit | 25629-1-AP | 1:1000 | WB |

**Table S3.** Antibodies used in IF and Co-IP analyses

| **Antibody** | **Manufacturer** | **Host** | **Catalog** | **Dilution** | **Assay** |
| --- | --- | --- | --- | --- | --- |
| p-MLKL | Cell Signaling Technology, USA | Rabbit | #91689 | 1:400 | IF |
| CD36 | Cell Signaling Technology, USA | Rabbit | #28109 | 1:200 | IF |
| NICD | Proteintech Group, USA | Rabbit | 20687-1-AP | 1:400 | IF |
| MAML1 | Novus Biologicals, USA | Rabbit | NBP1-83025 | 1:50 | IF |
| ASC | Cell Signaling Technology, USA | Rabbit | #67824 | 1:200 | IF |
| Caspase-1 | Cell Signaling Technology, USA | Rabbit | #24232 | 1:200 | IF |
| RIPK3 | Cell Signaling Technology, USA | Rabbit | #13526 | 1:50 | Co-IP |
| Caspase-1 | Cell Signaling Technology, USA | Rabbit | #24232 | 1:50 | Co-IP |
| ASC | Cell Signaling Technology, USA | Rabbit | #67824 | 1:50 | Co-IP |
| NLRP3 | Cell Signaling Technology, USA | Rabbit | #15101 | 1:50 | Co-IP |
