# Supplementary material for: CD36 Regulates PANoptosis in Diabetic Retinopathy via the NOTCH/MAML Pathway
Source: J Diabetes Res. 2026 Jul 24;2026:9324498. doi: 10.1155/jdr/9324498 (PMC13397476; doi:10.1155/jdr/9324498)
Supplement: Supplementary file 5 — Supporting Information 5 Figure S3: Uncropped Western blot membranes corresponding to Figure 1E of the manuscript. Molecular weight markers were added, and the expected positions of the proteins were indicated. The red box indicates the region used in the final figure. Figure S4: Uncropped Western blot membranes corresponding to Figure 3D of the manuscript. Molecular weight markers were added, and the expected positions of the proteins were indicated. The red box indicates the region used in the final figure. Figure S5: Uncropped Western blot membranes corresponding to Figure 4F of the manuscript. Molecular weight markers were added, and the expected positions of the proteins were indicated. The red box indicates the region used in the final figure. Figure S6: Uncropped Western blot membranes corresponding to Figure 6E of the manuscript. Molecular weight markers were added, and the expected positions of the proteins were indicated. The red box indicates the region used in the final figure. Figure S7: Uncropped Western blot membranes corresponding to Figure 7E of the manuscript. Molecular weight markers were added, and the expected positions of the proteins were indicated. The red box indicates the region used in the final figure. Figure S8: Uncropped Western blot membranes corresponding to Figure 8E of the manuscript. Molecular weight markers were added, and the expected positions of the proteins were indicated. The red box indicates the region used in the final figure. [file JDR-2026-9324498-s003.zip › Supplemental Material.docx]

**Supplemental Material**

**CD36 Regulates PANoptosis in Diabetic Retinopathy via the NOTCH/MAML Pathway**

Rongfeng Dai^*^, Yu Qian, Siqi Liu, Yuqing You, Yan Han, Yi Fang

Dear editor:

Thank you for your feedback. Please refer to the attachment for the original uncropped WB membranes.

**Figure S3.** Uncropped Western blot membranes corresponding to **Figure 1E** of the manuscript. Molecular weight markers were added, and the expected positions of the proteins were indicated. The red box indicates the region used in the final figure.

**Figure S4.** Uncropped Western blot membranes corresponding to **Figure 3D** of the manuscript. Molecular weight markers were added, and the expected positions of the proteins were indicated. The red box indicates the region used in the final figure.

**Figure S5.** Uncropped Western blot membranes corresponding to **Figure 4F** of the manuscript. Molecular weight markers were added, and the expected positions of the proteins were indicated. The red box indicates the region used in the final figure.

**Figure S5.** Uncropped Western blot membranes corresponding to **Figure 4F** of the manuscript. Molecular weight markers were added, and the expected positions of the proteins were indicated. The red box indicates the region used in the final figure.

**Figure S6.** Uncropped Western blot membranes corresponding to **Figure 6E** of the manuscript. Molecular weight markers were added, and the expected positions of the proteins were indicated. The red box indicates the region used in the final figure.

**Figure S6.** Uncropped Western blot membranes corresponding to **Figure 6E** of the manuscript. Molecular weight markers were added, and the expected positions of the proteins were indicated. The red box indicates the region used in the final figure.

**Figure S7.** Uncropped Western blot membranes corresponding to **Figure 7E** of the manuscript. Molecular weight markers were added, and the expected positions of the proteins were indicated. The red box indicates the region used in the final figure.

**Figure S7.** Uncropped Western blot membranes corresponding to **Figure 7E** of the manuscript. Molecular weight markers were added, and the expected positions of the proteins were indicated. The red box indicates the region used in the final figure.

**Figure S8.** Uncropped Western blot membranes corresponding to **Figure 8E** of the manuscript. Molecular weight markers were added, and the expected positions of the proteins were indicated. The red box indicates the region used in the final figure.

**Figure S8.** Uncropped Western blot membranes corresponding to **Figure 8E** of the manuscript. Molecular weight markers were added, and the expected positions of the proteins were indicated. The red box indicates the region used in the final figure.
